# SNAI1 promotes epithelial-mesenchymal transition and maintains cancer stem cell-like properties in thymic epithelial tumors through the PIK3R2/p-EphA2 Axis

**DOI:** 10.1186/s13046-024-03243-0

**Published:** 2024-12-19

**Authors:** Haoran E, Lei Zhang, Zhenhua Yang, Long Xu, Tao Wang, Junhong Guo, Lang Xia, Juemin Yu, Heyong Wang, Yunlang She, Junqi Wu, Yue Zhao, Chang Chen, Deping Zhao

**Affiliations:** 1 Department of Thoracic Surgery, Shanghai Pulmonary Hospital, School of Medicine, Tongji University, Shanghai, 200443 China; 2 Department of Thoracic Surgery, Ningbo No.2 Hospital, Ningbo, 315000 China; 3 Department of Pathology, Shanghai Pulmonary Hospital, School of Medicine, Tongji University, Shanghai, 200433 China; 4 Central Laboratory, Shanghai Pulmonary Hospital, School of Medicine, Tongji University, Shanghai, 200433 China

**Keywords:** Thymic epithelial tumors, SNAI1, Epithelial-mesenchymal transition, Cancer stem cells, Phosphoinositide-3-kinase regulatory subunit 2 (PIK3R2), Phosphorylated EPH receptor A2 (p-EphA2)

## Abstract

**Background:**

Thymic epithelial tumors (TETs) are infrequent malignancies that arise from the anterior mediastinum. Therapeutic options for TETs, especially thymic carcinoma (TC), remain relatively constrained. This study aims to investigate the oncogenic hub gene and its underlying mechanisms in TETs, as well as to identify potential therapeutic targets.

**Methods:**

Weighted gene co-expression network analysis (WGCNA) and differential gene expression (DEG) analysis were utilized to identify significant oncogenes using The Cancer Genome Atlas (TCGA) database. LASSO logistic regression analysis was performed to assess the association between hub genes and clinical parameters. The influence of the hub gene on promoting epithelial-mesenchymal transition (EMT), tumor progression, and regulating cancer stem cell-like properties was assessed both in vitro and in vivo. Single-cell RNA sequencing (scRNA-seq) was utilized to analyze the alterations in the tumor and its microenvironment following the administration of the hub gene’s inhibitor. Multiplex immunohistochemistry (mIHC) was employed to validate the results. The potential mechanism was further elucidated through the utilization of Cleavage Under Targets and Tagmentation (CUT&Tag), RNA-sequencing, chromatin immunoprecipitation (ChIP), CUT&RUN, luciferase reporter assay, co-immunoprecipitation (Co-IP), mass spectrometry (MS) and phosphoproteomic assays.

**Results:**

SNAI1 was identified as a hub transcription factor for TETs, and its positive correlation with the invasiveness of the disease was confirmed. Subsequent experiments revealed that the upregulation of SNAI1 augmented the migration, invasion, and EMT of TET cell lines. Furthermore, we observed that the overexpression of SNAI1 sustained cancer stem cell-like properties. ScRNA-seq demonstrated that the use of a SNAI1 inhibitor inhibited the transition of macrophages from M1 to M2 phenotype, a finding further validated by multiplex immunohistochemistry (mIHC). Phosphoinositide-3-kinase regulatory subunit 2 (PIK3R2) was identified as one of the downstream targets of SNAI1 through CUT&Tag and RNA-sequencing, a finding validated by ChIP-qPCR, CUT&RUN-qPCR, luciferase reporter and immunofluorescence assays. Co-IP, MS and phosphoproteomic assays further confirmed that PIK3R2 directly interacted with phosphorylated EphA2 (p-EphA2), facilitating downstream GSK3β/β-catenin signaling pathway.

**Conclusion:**

The tumorigenic role of SNAI1 through the PIK3R2/p-EphA2 axis was preliminarily validated in TETs. A potential therapeutic strategy for TETs may involve the inhibition of SNAI1.

**Supplementary Information:**

The online version contains supplementary material available at 10.1186/s13046-024-03243-0.

## Background

Thymic epithelial tumors (TETs) originate from the thymus. Despite being one of the frequently encountered neoplasms in the anterior mediastinum, the incidence rate of TETs is relatively low, approximately 1.5 cases per million [1–3]. Recent advances in multi-omics technologies have enabled researchers to gain a more profound understanding of TETs. For example, researchers have identified four distinct molecular subtypes of TETs [4]. The molecular subgrouping has been demonstrated to be closely related to the clinical characteristics of the disease. A subsequent study integrated data from genomics, transcriptomics, epigenetics, and proteomics sequencing, providing a further characterization of the genomic landscape of TETs [5]. The characteristics of the tumor microenvironment were also elucidated using mass cytometry and single-cell RNA sequencing [6]. Unfortunately, although researchers have acquired preliminary insights into TETs, the exploration of potential treatment targets and drug development remains in its early stages. Recent clinical trials have only provided support for the efficacy of sunitinib and lenvatinib, both multi-tyrosine kinase receptor inhibitors, in the context of chemotherapy-refractory TETs [7, 8]. The feasibility of utilizing pembrolizumab and nivolumab exclusively as non-first-line therapies for recurrent or advanced TETs has been demonstrated [9–11]. Additional studies are still warranted to delve into the tumorigenic mechanisms and identify potential therapeutic targets for TETs.

Epithelial-mesenchymal transition (EMT) is a biological process characterized by the transformation of epithelial cells into mesenchymal cells. It plays a crucial role in various physiological processes, including tissue remodeling, wound healing, and tumorigenesis [12]. Accompanied by characteristic morphological changes, the genetic, physiological, and metabolic alterations collectively contribute to the tumorigenic transition of tumor cells [13, 14]. It has also been validated to be closely associated with the acquisition of cancer cell stemness [15]. The Snail1 protein, encoded by SNAI1 gene, is a well-studied transcription factor that directly promotes EMT, hence being recognized as one of the EMT markers [16]. It can directly bind to the promoter regions of numerous downstream target genes. Due to Snail1’s pivotal role in EMT and the maintenance of cancer stemness, it has also been considered as a promising therapeutic target for drug development [17]. However, the tumorigenic function of Snail1 in TETs has not been well validated thus far. The fundamental effects of Snail1 in promoting EMT and facilitating the acquisition of cancer stemness in TETs remain to be elucidated. Additionally, it is also intriguing to investigate whether Snail1 can be identified as a potential target for future therapeutic interventions in TETs.

In this study, we identified SNAI1 as the central transcription factor promoting the tumorigenesis of TETs through comprehensive analysis of bioinformatic data from the TCGA database. The function of SNAI1 in promoting EMT and sustaining cancer stem cell-like properties was validated both in vitro and in vivo. Furthermore, our findings demonstrated that SNAI1 regulates macrophage polarization and thus stimulates tumor stemness. Investigation into the underlying mechanism revealed that the oncogenic role of SNAI1 operates through a PIK3R2/p-EphA2 axis. Taken together, these findings illuminate novel insights into the understanding of the progression of TETs.

## Materials and methods

### Human specimens, mice, and study approval

This study comprised four clinical naive TET samples along with their corresponding adjacent normal samples. Written informed consents were obtained from all the patients. Five- to six-week-old male wild-type BALB/c nude mice were acquired from the Animal Laboratory of Shanghai Pulmonary Hospital. The study protocol received approval from the Institutional Review Board of Shanghai Pulmonary Hospital.

### Cell lines and cell culture

The Ty82 cell line, derived from an undifferentiated thymic carcinoma sample (JCRB1330), was generously provided by Prof. Ze-Hong Miao from the Shanghai Institute of Materia Medica, Chinese Academy of Sciences. The IU-TAB-1 cell line, derived from a type AB thymoma sample, was acquired from the Applied Biological Materials Inc. (abm, BC, Canada). Ty82 cells are cultured in RPMI 1640 medium (Procell, Wuhan, China), and IU-TAB-1 cells are maintained in DMEM medium (Procell). All medium contained 10% fetal bovine serum (FBS; Procell) and 1% penicillin-streptomycin (Hyclone, cytiva, MA, USA). All cellular cultures were upheld in a humidified incubation chamber with 5% CO2 at 37 °C and regularly tested for mycoplasma contamination.

### WGCNA, DEG, and LASSO-logistic analyses

The open-access RNA-sequencing data and clinical characteristics of TET patients were retrieved from TCGA website. Following the removal of genes lacking sequencing data, a transformed transcripts per kilobase million (TPM) matrix comprising 22,273 genes was included in the weighted gene co-expression network analysis (WGCNA). Subsequently, we calculated the adjacency using the adjacency function with a weighting coefficient (β) set to 6, created the Topological Overlap Matrix (TOM) using TOMsimilarity, generated the gene tree through hclust with method = “average”, and performed dynamic tree cutting with a minimum module size of 50. The eigengenes of each module were subsequently correlated with clinical information, including histological types and pathological stages. Differential gene expression (DEG) analysis was also conducted based on the TCGA-THY database. A TPM matrix comprising 56,536 genes were included in the DEG analysis using limma analysis. Genes of interest, along with their expression matrix filtered from the TCGA-THY database, were input into a Least Absolute Shrinkage and Selection Operator (LASSO) regression model. A tuning parameter (λ) was introduced into the regression model to impose a penalty on the coefficients of the genes. A predictive signature was formulated by combining these selected genes in a linear manner with their respective weighted coefficients. Utilizing the signature, risk scores were computed for each patient according to the expression profiles of the selected genes. Subsequently, risk scores were compared among distinct clinical status groups.

### Immunohistochemistry analysis

Immunohistochemistry (IHC) analysis was performed using 3 clinical TET samples diagnosed as thymic carcinoma, along with their matched adjacent normal samples. Additionally, a tissue microarray, acquired from Bioaitech (Xi’an, China, Cat. no. I054Th01) and comprising 54 samples, was also included in the analysis. The tissue microarray comprised 46 TET samples, categorized as Type A (6), Type AB (22), Type B1 (6), Type B2 (6), Type B3 (2), and TC (4), along with 2 thymic hyperplasia samples and 6 normal thymic samples. The IHC procedure was conducted by Servicebio Technology (Wuhan, China) using corresponding antibodies. Staining intensity was initially assessed and scored as follows: no staining (0), light yellow (1), brown-yellow (2), and brown (3). The positive cell ratio was assessed as follows: 1, 0%∼25%; 2, 26%∼50%; 3, 51%∼75%; 4, more than 75%. The ultimate staining score was calculated by multiplying the staining intensity score with the positive cell ratio score.

### Plasmid construction, Lentivirus virus production and infection, and application of small-molecule inhibitors

The pSLenti-EF1-CMV-EGFP-P2A-Puro-WPRE vector designed for overexpressing SNAI1 in human TET cell lines was constructed. For SNAI1 knockdown, the pSLenti-U6-CMV-EGFP-F2A-Puro-WPRE vector was employed to construct short hairpin RNA (shRNA) plasmids. For SNAI1 knockdown, the pSLenti-U6-CMV-EGFP-F2A-Puro-WPRE vector was employed to construct short hairpin RNA (shRNA) plasmids. For PIK3R2 knockdown, the pSLenti-U6-CMV-EGFP-F2A-Puro-WPRE vector was utilized to construct short hairpin RNA (shRNA) plasmids. All these plasmids were procured from OBiO Technology (Shanghai, China).

TET cells were seeded into 6-well plates at a density of 2 × 10^5 cells per well. After 24 h, when the cells reached approximately 70% confluence, two solutions were prepared for transfection. Solution 1 contained 7.5 µg of psPAX2, 2.5 µg of pMD2.G, and 10 µg of shRNA, all dissolved in 2.5 ml of plain DMEM. Solution 2 was prepared by dissolving 60 µL of Lipofectamine 2000 in 2.5 ml of plain DMEM. The two solutions were incubated together for 25 min at room temperature before being applied to the TET cells for transfection. Twelve hours post-transfection, the cell media was replaced with fresh complete media and collected after 48 h. The collected media samples were pooled, and viral particles were harvested by filtering the pooled media through a 0.45 μm PES filter. The virus was then aliquoted and stored at -80 °C.

For infection, TET cells at 20–30% confluence in each well of a standard 6-well plate were incubated for 12 h with a mixture containing virus at a multiplicity of infection (MOI) of 3 to 5, 5 µl/ml polybrene, and fresh complete media. After incubation, the cells were maintained in fresh complete media for an additional 24 h before proceeding with downstream experiments.

Two small-molecule inhibitors were utilized in this study. Rosiglitazone, known for its inhibitory effect on Snail1 [18, 19], was obtained from MedChemExpress (NJ, USA). TET cells were incubated with 10 µM rosiglitazone for 24 h. Dasatinib, which targets EphA2 phosphorylation at Ser897 [20], was acquired from ApexBio (TX, USA). TET cells were incubated with 100 nM dasatinib for 48 h.

### Quantitative real-time polymerase chain reaction (qRT-PCR) analysis

Total RNA from TET cells was extracted using TRIzol reagent (Thermo Scientific, MA, USA). Following quantification with a Nanodrop 1000 spectrophotometer (Thermo Scientific), cDNA was reverse-transcribed and synthesized using the Mastercycler Nexus (Eppendorf, Hamburg, Germany). Gene expression levels were computed using the 2^(-ΔΔCt) method. The primer sequences for SNAI1, CD133, CD44, PIK3R2, GAPDH, along with the designed primers for the ChIP-qPCR assay, are provided in Supplementary Table S1.

### Western blot analysis

Total protein was prepared using a protein extraction kit (Beyotime, Shanghai, China). After protein quantification using Coomassie Brilliant Blue (Servicebio), equal amounts of protein were separated through Sodium Dodecyl Sulfate Polyacrylamide Gel Electrophoresis (SDS-PAGE) using gels from Epizyme Biotech (Shanghai, China). Afterwards, proteins were transferred onto a polyvinylidene fluoride (PVDF) membrane or nitrocellulose (NC) membrane. After blocking with a blocking buffer (Epizyme Biotech), membranes were incubated overnight at 4℃ with primary antibodies, followed by a one-hour incubation with secondary antibodies. All the antibodies used in this study are listed in Supplementary Table S2.

### Cell proliferation assay

For the cell counting kit-8 (CCK-8) assay, TET cells were seeded into 96-well plates at a density of 5 × 10^3 cells per well for cultivation. Subsequently, the culture medium was replaced with a medium containing 10% CCK-8 reagent (Elabscience, Wuhan, China) at various time points (24, 48, 72, and 96 h), and incubated for 2 h. The absorbance of each sample was then quantified at 450 nm. For the clonal assay, 2 × 10^3 cells were plated into each well of 6-well plates. Following 14 days of cultivation, the cells were rinsed with PBS, fixed using 4% paraformaldehyde, and stained with a crystal violet solution (Solarbio, Beijing, China).

### Cell migration and invasion assay

For the cell migration assay, 1 × 10^5 cells were suspended in 200 µL of FBS-free medium and placed in the upper chamber of the transwell filter. The lower chamber of the filter was filled with 600 µL of 10% FBS medium. After 48 h of culture, cells migrating through the filter were fixed with 4% paraformaldehyde and stained with a crystal violet solution (Solarbio). For the cell invasion assay, the front side of the filter was pre-coated with Matrigel matrix. Subsequently, 1 × 10^5 cells were placed in the upper chamber, and cells invading the filter were fixed and stained.

### Flow cytometry assay

Single-cell suspensions of TET cells were obtained and resuspended for subsequent experiments. For the cell apoptosis assay, the Annexin V-APC/PI Apoptosis Kit (Elabscience) was employed. For CSC marker staining, specific antibodies were sequentially incubated as follows: PE anti-human/mouse CD44 (Elabscience, Cat. no., E-AB-F1100D), oval cell marker antibody (OV-6, Santa Cruz, Cat. no., sc-101863), and APC mouse IgG2a, κ isotype control (Elabscience, Cat. no., E-AB-F09802E). Results from the flow cytometry assay were analyzed using FlowJo software (BD, OR, USA).

### CSC spheroid formation assay

DMEM/F-12 culture medium (Gibco, Thermo Scientific) supplemented with growth factors including epidermal growth factor (EGF) and fibroblast growth factor (FGF) was prepared. A total of 2 × 10^5 TET cells were suspended in the medium and plated into each well of ultra-low attachment 6-well plates (Corning, NY, USA) for 10–12 days. The morphology of the CSC spheres was observed using inverted microscopy. The detailed protocol for the spheroid formation assay is provided in our previous publications [21, 22].

### In vivo tumor metastasis assay

The 5- to 6-week-old male wild-type BALB/c nude mice were maintained in a laminar airflow cabinet within the hospital’s animal laboratory. TET cells (Ty82, 5 × 10^5/100µL) were injected into the left ventricle of the nude mice and allowed to metastasize for nearly a month. To assess the metastasis status of the mice from different groups, in vivo imaging was conducted using the indiGo software (Berthold Technologies, Germany) to detect the green fluorescent protein expressed by the infected lentivirus. The specific procedure was referenced from the study conducted by Franke-Fayard et al. [23].

### PDX model establishment and sequencing of scRNA-seq

Surgical specimens from a pathologically diagnosed thymic squamous cell carcinoma were obtained and dissected into small fragments. These samples were subsequently subcutaneously implanted into the right flank of 5- to 6-week-old BALB/c nude mice. After allowing the tumors to grow for nearly 2 weeks, mice successfully inoculated with tumors (with a tumor size ranging from 100 to 150 mm^3^) were randomly assigned to either the treatment group or the control group. A total of 4 mice, with 2 in each group, were included. The treatment group received intraperitoneal injections of 100 µL PBS containing 10 mg/kg rosiglitazone once every 2 days for a duration of 14 days. Subsequently, the specimens were harvested and subjected to single-cell RNA sequencing (scRNA-seq) analysis.

The scRNA-seq was performed by BGI Genomics (Shenzhen, China). All specimens were dissociated into single cells, resuspended, and loaded for further analysis. The human genomes were aligned to the Ensembl release 104 genome reference. The mouse genomes were aligned to the GCF_000001635.26_GRCm38.p6 genome reference.

### Multiplex immunohistochemistry assay

The mIHC assay was conducted by Servicebio Technology using the tissue microarray (Bioaitech, Xi’an, China, Cat. no. I054Th01) consisting of 54 samples. The following antibodies were utilized in the assay: SNAI1 polyclonal antibody (proteintech, Cat. no., 13099-1-AP), anti-CD44 rabbit pAb (Servicebio, Cat. no., GB112054-100), anti-CD68 rabbit pAb (Servicebio, Cat. no., GB11067-100), anti-CD86 rabbit pAb (Servicebio, Cat. no., GB115630-100), and anti-CD206 rabbit pAb (Servicebio, Cat. no., GB115273-100). Digital images were scanned and visualized in SlideViewer software (3DHISTECH Ltd., Hungary).

### CUT&Tag and RNA sequencing

Cleavage under targets and tagmentation (CUT&Tag) sequencing was performed by OEBiotech (Shanghai, China). Two sets of Ty82 cells overexpressing SNAI1 were immobilized on concanavalin A-coated magnetic beads. After permeabilization with digitonin, cells were sequentially incubated with the primary antibody (SNAI1, proteintech) and secondary antibody. Afterwards, hyperactive pA/pG-Tn5 transposon was incubated and bound to the secondary antibody. The transposon was then activated and involved in DNA fragmentation. Tumor cells were subsequently cleaved, and DNA fragments were extracted for the subsequent library preparation and sequencing. RNA sequencing (RNA-seq) was conducted by Personalbio Technology (Shanghai, China). Two sets of Ty82 cells were subjected to RNA sequencing, with one group overexpressing SNAI1 and the other group maintaining normal expression levels. Each group comprised three samples. The analysis of DEGs was also performed using the limma analysis method.

### ChIP-qPCR assay

The Chromatin Immunoprecipitation (ChIP) assay was performed using the SimpleChIP Plus Enzymatic Chromatin IP Kit (Cell Signaling Technology, MA, USA, Cat. no. 9005). The antibody used in the ChIP process was SNAI1 (Cell Signaling Technology, Cat. no. 3879). The quantification of the purified DNA sample was conducted using the qRT-PCR method. The binding sites were identified based on the predicted sequence generated using the JASPAR database. The primers were then synthesized according to the NCBI Primer-BLAST report.

### Luciferase reporter assay

A dual-luciferase reporter gene assay was used to assess the effect of the transcription factor SNAI1 on PIK3R2 promoter activity. The plasmid was constructed by OBiO Technology (Shanghai, China). The main experimental steps include plasmid transfection of cells, dual-reporter gene assay, and statistical analysis. Ty82 cells were seeded into a 96-well plate at 70% confluence. After 24 h, the luciferase reporter gene plasmid and the transcription factor gene plasmid were transfected, with 6 replicates per sample. When transfecting plasmids, the ratio per well was Firefly luciferase vector: Renilla luciferase vector: transfection reagent = 0.1 µg: 0.005 µg: 0.25 µl. Luciferase signals were measured 48 h post-transfection using the Dual-Luciferase Reporter Assay System (Promega, Beijing, China; Cat. no. E1910).

### CUT&RUN assay

The CUT&RUN assay was performed on IU-TAB-1 cells using the CUT&RUN Assay Kit (Cell Signaling Technology, MA, USA; Cat. No. 86652). The antibody used in the CUT&RUN process was SNAI1 (Cell Signaling Technology, Cat. no. 3879). This assay was conducted following the manufacturer’s protocol, using IU-TAB-1 cell lines. The quantification of the purified DNA sample was conducted using the qRT-PCR method.

### Immunofluorescence colocalization assay

The immunofluorescence colocalization assay was performed using IU-TAB-1 cells overexpressing SNAI1 and corresponding control cells. The assay utilized the following antibodies: Snail (C15D3) Rabbit mAb (Cell Signaling Technology, Cat. no., 3879) and Anti-PI 3 Kinase p85 beta antibody (abcam, Cat. no., ab180967). Laser confocal microscopy (Leica, Germany) was used for imaging. Digital images were visualized in LAS X microscope software platform (IL, USA).

### Co-IP, MS and phosphoproteomic assays

The co-immunoprecipitation (Co-IP) assay was conducted using the Immunoprecipitation Kit with Protein A + G Magnetic Beads (Beyotime, Cat. no. P2179S). Briefly, adherent TET cells were lysed using lysis buffer containing the protease inhibitor cocktail. The primary antibody (PIK3R2, Abcam, UK, Cat. no. ab180967) was bound to the Protein A + G Magnetic Beads. The samples were incubated with the magnetic beads, and the extracted protein was eluted and utilized for subsequent analysis. The proteins obtained through Co-IP were visualized using a silver staining kit (Sangon Biotech, Cat. no. C500021, Shanghai, China). Mass spectrometry (MS) and phosphoproteomic analysis were conducted by Deepkinase (Beijing, China). The protein that directly interacts with PIK3R2 was identified using mass spectrometry (MS). Phosphoproteomic analysis was also performed to investigate the direct interaction between PIK3R2 and phosphorylated proteins.

### Statistical analysis

Continuous variables were expressed as the median with interquartile range (IQR) and were compared using the non-parametric Mann-Whitney U test or Kruskal-Wallis one-way ANOVA. Spearman’s correlation analysis was employed to assess the relationship between two variables. ImageJ software (LOCI, WI, USA) was utilized for the visualization and interpretation of imaging data. Statistical analyses were conducted using SPSS (version 26, IBM Corp., NY, USA). Column charts and line charts were generated using GraphPad Prism (version 9.0, Dotmatics, CA, USA). Venn diagrams were generated using an online tool from Bioinformatics & Evolutionary Genomics (Belgium). WGCNA analysis, DEG analysis, LASSO-logistic regression, ROC curves, scRNA-seq analysis, and KEGG analysis were conducted using the “ggplot2”, “WGCNA”, “ComplexHeatmap”, “limma”, “glmnet”, “survival”, “pROC”, “Seurat”, “SingleR”, “DoubletFinder”, “dplyr”, “harmony”, “clusterProfiler”, “enrichplot”, “inferCNV”, “FeaturePlot”, “monocle”, “CytoTRACE”, “CellChat” and “GeneSwitches” packages in R software (version 4.1.1, https://cran.r-project.org). All figures were prepared using Adobe Illustrator (Adobe Inc., CA, USA). All *p*-values were two-sided with a statistical significance level set at less than 0.05. In all the results, * indicates *p* < 0.05, ** indicates *p* < 0.01, *** indicates *p* < 0.001, and ns indicates not statistically significant.

## Results

### SNAI1 is identified as the pivotal transcription factor for TETs through the application of WGCNA and DEG analyses

**Fig. 1:**
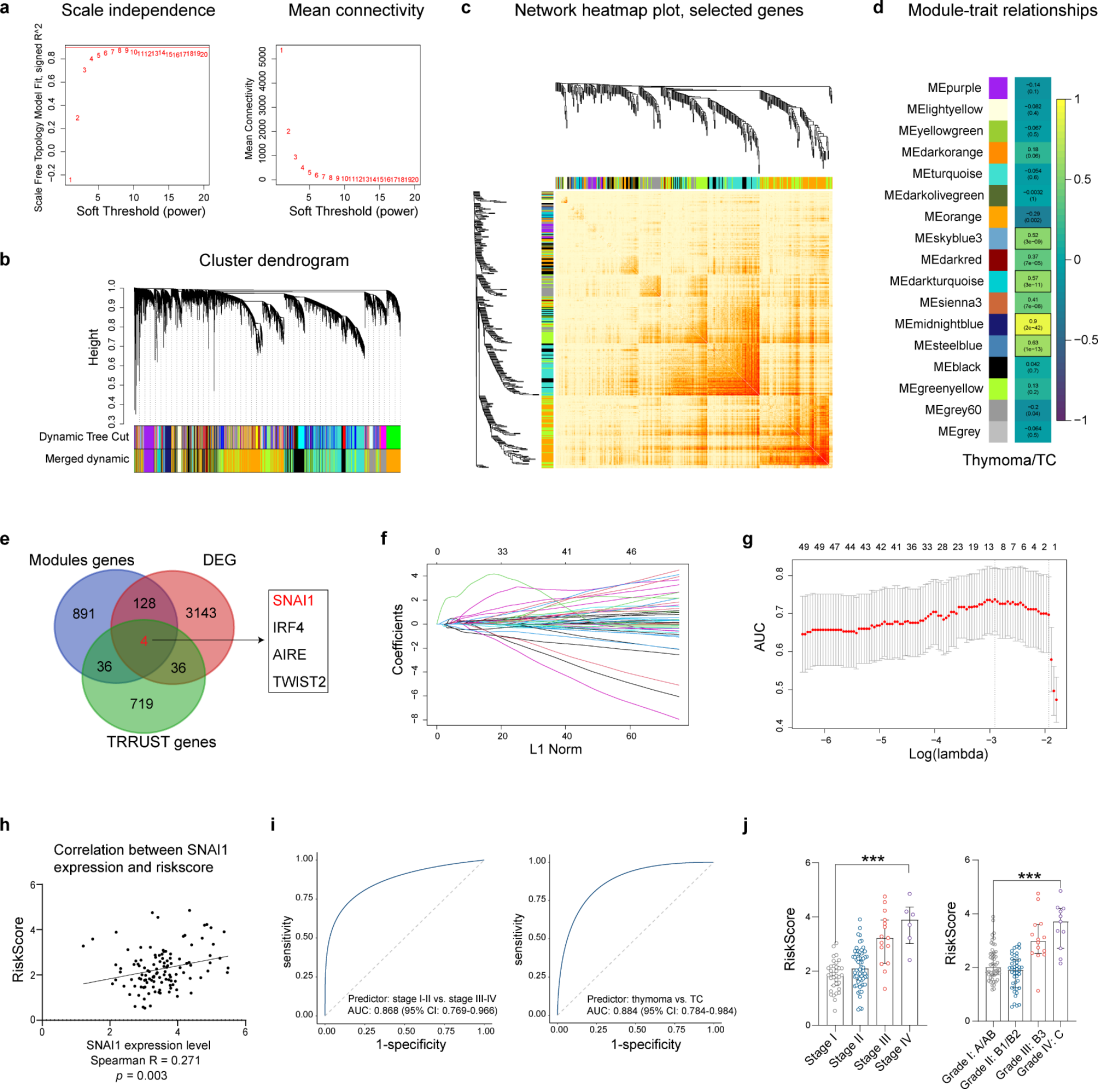
Identification of the hub transcription factor through WGCNA and DEG analyses. **a**. Exploration of the network topology under different soft-thresholding powers (weighting coefficient, *β*). The x-axis denotes distinct soft-thresholding powers, while the y-axis illustrates the correlation coefficient between log (k) and log [P(k)]. The red line signifies a correlation coefficient of 0.9. Average network connectivity under different weighting coefficients. The average network connectivity under different weighting coefficients is also depicted. **b**. Dendrograms illustrating clustering of all DEGs, with dissimilarity based on topological overlap, along with assigned module colors. In total, 17 co-expression modules were constructed and are represented by different colors. **c**. Heatmap plot illustrating the gene network, displaying the Topological Overlap Matrix (TOM) among all Differentially Expressed Genes (DEGs) in the analysis. Darker red indicates higher overlap, while lighter colors indicate lower overlap. Module assignment genes and dendrogram are presented along the top and left sides. **d**. The figure illustrates module-trait associations, where the column corresponds to the trait (thymoma or TC), and each row represents a Module Eigengene (ME). The numbers in the rectangles denote the correlation coefficient, with the corresponding *p* value shown in brackets. The table is color-coded based on the correlation, as indicated by the color legend. **e**. The Venn diagram shows the intersection of the significant Modules genes, DEGs, and transcription factors from the TRRUST database. **f**. The LASSO coefficient spectrum of 12 genes is depicted, presenting a distribution map based on a logarithmic (λ) sequence. **g**. The figure displays the partial likelihood deviance for varying numbers of variables as revealed by the LASSO regression model. The red dots signify the partial likelihood deviance values, while the grey lines represent the standard error (SE). The two vertical dotted lines on the left and right, respectively, indicate optimal values based on the minimum criteria and 1-SE criteria. **h**. The figure depicts the correlation analysis between the expression level of SNAI1 and the risk-scores generated by the LASSO regression model. **i**. The figure displays ROC curves for predicting pathological stages and pathological subtypes using the risk-scores. **j**. The columnar distributions of risk-scores among different stages and pathological subtypes. *** denotes *p* < 0.001

A total of 119 patients from the TCGA database with RNA-seq data were included in the WGCNA (Figure S1a). The weighting coefficient, denoted as *β*, was selected as 6 to construct the co-expression network (Fig. 1a). A hierarchical clustering tree was generated through hierarchical clustering utilizing dissTOM. The minimum number of genes per module was defined as 50 to discern the key cluster. After calculate the MEs (Figure S1b) and merging the two modules with a correlation coefficient exceeding 0.75, the final number of the acquired modules amounted to 17 (Fig. 1b). The Spearman correlation coefficients were computed between the MEs and the corresponding clinical information to discern associations between specific modules and clinical traits. Conclusively, we scrutinized the modules that exhibited a significantly positive association (*R* > 0.5, *p* < 0.05) with histological type, categorized as binary (thymoma or TC) (Fig. 1c,d, Figure S1c–e). A total of 4 modules, encompassing 1,059 genes, were identified as hub module genes (Fig. 1e). Subsequently, we performed a DEG analysis comparing TET tissues (*n* = 119) with normal adjacent tissues (*n* = 2). A total of 3,311 genes were identified as DEGs. Additionally, we retrieved a compilation of 795 transcription factors from the TRRUST database. The intersection of these three gene sets comprised SNAI1, IRF4, AIRE, and TWIST2 (Fig. 1e). To evaluate the protein expression of SNAI1 in TETs, immunohistochemical staining was conducted on 3 samples diagnosed as TCs from our institution. Quantitative analysis of the staining intensity and percentage unveiled an apparent upregulation in the expression of SNAI1 in TC tissues as compared to adjacent normal tissues (Figure S1f-g).

To further investigate the correlation between the expression level of SNAI1 and the invasiveness of TETs, LASSO-logistic regression analysis was performed. Two hundred genes from the Hallmark gene set related to epithelial-mesenchymal transition (Molecular Signatures Database) were selected to formulate the model. The clinical traits examined encompassed pathological stages, categorized dichotomously as either stage I-II or III-IV. Ultimately, a predictive model comprising 12 genes was developed (Fig. 1f-g). The expression of SNAI1 was assessed to be positively correlated with the risk scores generated through LASSO-logistic analysis (*R* = 0.271, *p* = 0.003) (Fig. 1h). Furthermore, the risk scores demonstrated notable effectiveness in predicting the pathological stages and histological types of TETs (Fig. 1i). These scores were also found to be correlated with the invasiveness of the disease (Fig. 1j). Collectively, these data demonstrate that SNAI1 serves as the pivotal transcription factor for TETs.

### SNAI1 plays a crucial role in the maintenance of the invasiveness of TETs

**Fig. 2:**
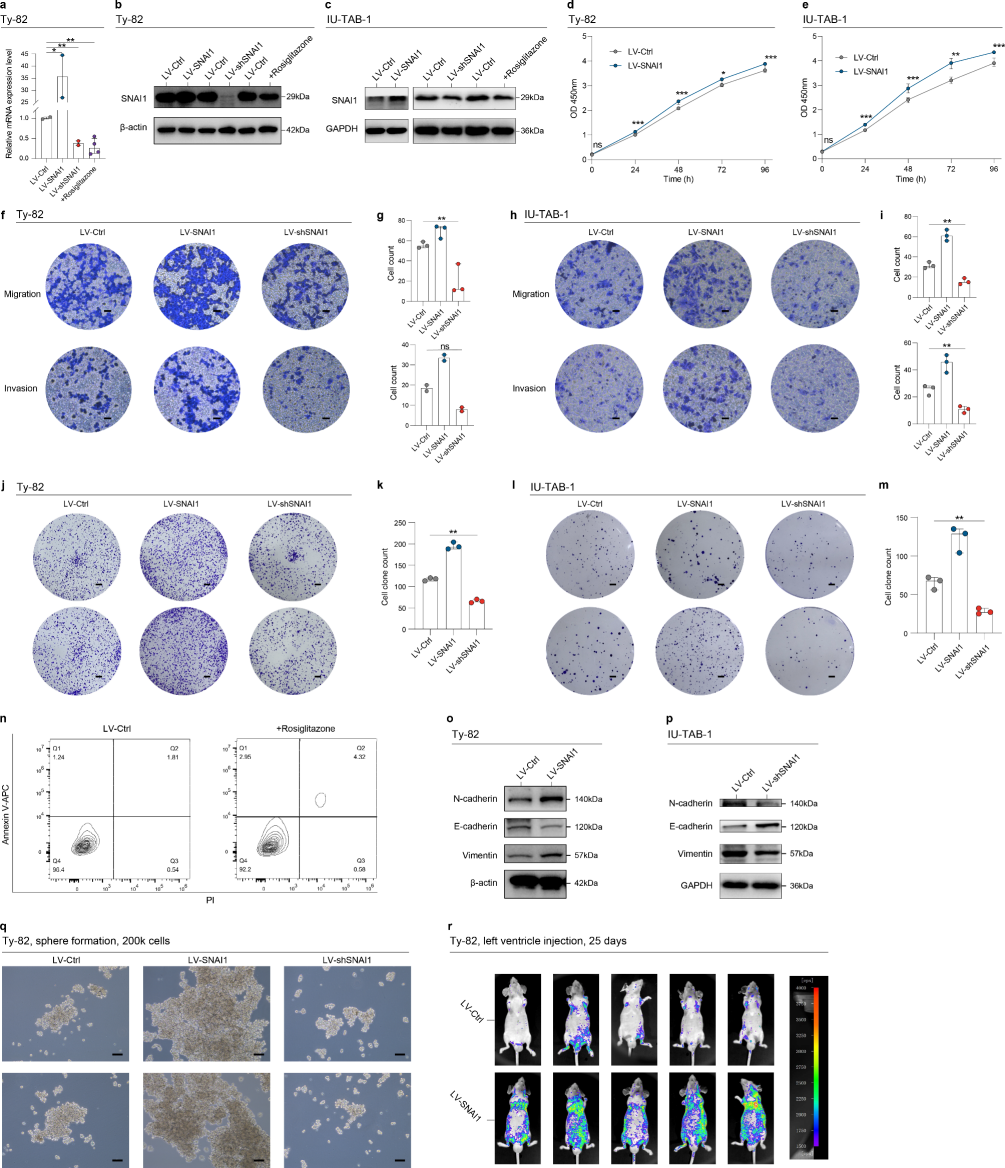
Validation of the oncogenic effect of SNAI1 both in vitro and in vivo. **a**. qRT-PCR reveals the relative mRNA expression levels in Ty82 cells under various conditions, including those overexpressing SNAI1 (LV-SNAI1), suppressing SNAI1 (LV-shSNAI1), treated with the SNAI1 inhibitor (Rosiglitazone), and the control group. **b**-**c**. Western blot analysis depicting the protein expression levels of SNAI1 in TET cells under different experimental conditions. **d**-**e**. The impact of SNAI1 overexpression on the proliferation of TET cells was assessed using the CCK-8 assay. Absorbance at 450 nm was measured at various time points. **f**-**i**. Transwell assays were employed to evaluate the impact of SNAI1 overexpression or suppression on the in vitro migration and invasion ability of TET cells. Representative micrographs and quantification of cells attached to the lower surface of the chamber are presented (Scale bars: 200 μm). **j**-**m**. The clone assay was utilized to assess the influence of SNAI1 on the in vitro proliferation of TET cells. Representative micrographs are presented (Scale bars: 2 mm). The quantifications of cell clone spheres are presented. **n**. The apoptosis assay was conducted using an Annexin V-APC/PI Apoptosis Kit in Ty82 cells treated with Rosiglitazone or the control group. **o**-**p**. Western blot analysis was performed to assess the protein expression levels of EMT markers, including N-cadherin, E-cadherin, and Vimentin, in TET cells transfected with LV-SNAI1 or LV-Ctrl. **q**. Representative images depict Ty82 cells transfected with LV-SNAI1, LV-shSNAI1, or LV-Ctrl grown as spheres (Scale bars: 200 μm). **r**. Ty82 cells transfected with LV-SNAI1 or LV-Ctrl were injected into the left ventricle of 5- to 6-week-old male wild-type BALB/c nude mice. Representative bioluminescent images depict TET cell metastases. * denotes *p* < 0.05, ** denotes *p* < 0.01, *** denotes *p* < 0.001, and ns denotes not statistically significant

To ascertain the oncogenic function of SNAI1 in TETs, we performed in vitro and in vivo validations. Initially, we established TET cell lines with varying expression levels of SNAI1 (Fig. 2a-c). A SNAI1 inhibitor, namely rosiglitazone [18, 19], was also employed. The overexpression of SNAI1 significantly enhanced the proliferation, migration, and invasion capabilities of TET cells, whereas the knockdown of SNAI1 reversed this effect (Fig. 2d-m). The administration of rosiglitazone also resulted in a higher percentage of apoptotic cells (Fig. 2n). Given that SNAI1 is a widely recognized marker for epithelial-mesenchymal transition, we also assessed the expression levels of other markers following the overexpression of SNAI1 in TET cells (Fig. 2o-p). Subsequently, TET cells were inoculated into the left ventricle of 5- to 6-week-old BALB/c nude mice, and we found that the overexpression of SNAI1 significantly induced tumor metastasis in vivo (Fig. 2r). To further assess the clinical correlation between SNAI expression and tumor invasiveness, IHC analysis was conducted utilizing a tissue microarray comprising 54 samples. The results indicated that the staining scores of SNAI1 were significantly higher in the Type B3 and TC samples, as well as in the advanced-stage samples (Figure S2a-b). The staining scores of pan-CK exhibited a similar trend to SNAI1, suggesting a correlation between SNAI1 expression and disease invasiveness (Figure S2c-d).

To further assess the impact of SNAI1 in sustaining cancer stem cell-like properties, a spheroid assay was conducted. TET cells were induced to form spheres in serum-free and growth factor-supplemented conditions. Quantitative real-time PCR analysis confirmed that the expression levels of cancer stem cell markers, including CD133 and CD44, in the spheres were significantly elevated compared to those in adherent cells (Figure S3a). The overexpression of SNAI1 significantly promoted the formation of a greater number of spheres, while the knockdown of SNAI1 attenuated this effect (Fig. 2q). The overexpression of SNAI1 in the spheres also led to an upregulation of the CSC markers (Figure S3b). OV6 has been identified as a recently discovered marker for cancer stem cells in our previous studies [21, 22]. It has been further validated to be closely associated with the invasiveness of TETs through the utilization of the tissue microarray (Figure S3c-d). The flow cytometry assay also unveiled a notable increase in the double-positive rate for OV6 and CD44 in the spheres with SNAI overexpression (Figure S3e). Together, these findings confirmed the pivotal role of SNAI1 in sustaining the stemness of TETs.

### SNAI1 inhibitor has the potential to reverse M1/M2-macrophage polarization and suppress tumor stemness

**Fig. 3:**
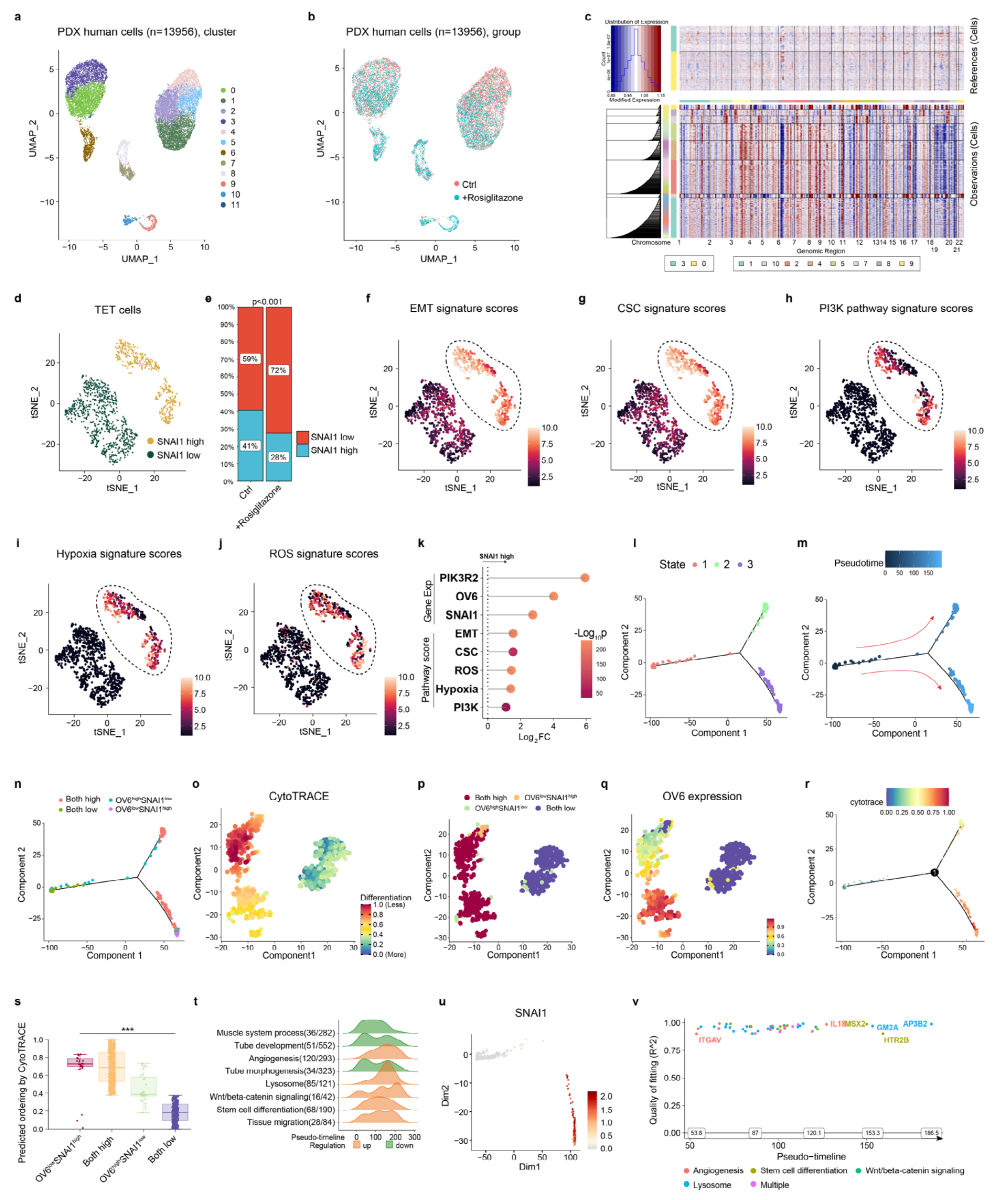
Evaluation of the impact of the application of the SNAI1 inhibitor on the TET cells through scRNA-seq. **a**-**b**. UMAP plot representing human cells (dots) derived from PDX models of thymic squamous cell carcinoma, with color-coding based on clusters and groups. **c**. Resuls of the inferCNV analysis of all the human cells. **d**-**e**. The expression level of SNAI1 across all tumor cells. **f**-**j**. The signature scores of critical biological pathways in all tumor cells. **k**. Geneset enrichment analysis (GSEA) showing normalized enrichment scores. **l**-**n**. Monocle plots demonstrating the differentiation trajectories based on monocle clusters, pseudotime, and gene expression status. **o**-**q**. t-SNE plot of tumor cells showing different CytoTRACE scores, subgroups, and different expression levels of OV6. **r**. Monocle plots demonstrating the differentiation trajectories based on CytoTRACE scores. **s**. Boxplots demonstrating CytoTRACE scores of different subgroups. **t**. Ridge plot demonstrating gene ontology pathways that were significantly altered across pseudotime. **u**. UMAP projections of expression levels for SNAI1. **v**. Geneswitches output showing the ordering of the top switching genes along the differentiation trajectory. *** denotes *p* < 0.001

The interactions between cancer stem cells and other immune cells in the microenvironment have sparked considerable interest among researchers [24]. Understanding the crosstalk between these two groups may contribute to the identification of potential therapeutic options. To further assess the impact of SNAI1 expression in primary TET cells on the tumor microenvironment, we subsequently conducted scRNA-seq analysis of the PDX models. The PDX models were established using resected surgical specimens obtained from a patient pathologically diagnosed with thymic squamous cell carcinoma. The PDX model, bearing established tumors, were randomly assigned to either the rosiglitazone group (10 mg/kg, intraperitoneal injection, once every 2 days) or the control group and underwent treatment for a duration of 14 days. We began by examining human cancer cells. A total of 13,956 human cells were obtained (Fig. 3a-b). To identify human TET cells, we performed inferCNV analysis. Clusters 3 and 0 were utilized as reference normal groups, representing the fibroblasts (Fig. 3c). Four clusters were identified as the epithelial cell population. Subsequently, we isolated all the cancer cells and conducted an analysis of SNAI1 expression levels within these cells. After administering rosiglitazone, a notable reduction was observed in the proportion of the group with high SNAI1 expression (Fig. 3d-e). We also developed signature models encompassing various biological markers, including those associated with EMT, cancer stemness, the PI3K pathway, hypoxia, and reactive oxygen species (ROS). Cancer cells exhibiting elevated SNAI1 expression levels were also identified as having significantly higher scores in these signature models (Fig. 3f-k). These results suggest that SNAI1 serves as a central regulator in these critical biological processes. To gain deeper insights into the differentiation trajectories of the cancer cells, we conducted Monocle and CytoTRACE analyses. All the cancer cells were divided into three states. Cells in states 2 and 3 exhibited high levels of SNAI1 expression. These cells also exhibited higher signature scores for critical biological processes (Figure S4a-d). The differentiation status of cancer cells was also closely related to SNAI1 expression (Figure S4e-i). To better understand the role of SNAI1 in maintaining cancer stemness, we included the CSC marker OV6 in the analysis. Most cells in states 2 and 3 exhibited high expression levels of both SNAI1 and OV6 (Fig. 3l-n). The differentiation status of cancer cells showed a close association with the expression levels of both SNAI1 and OV6 (Fig. 3o-s). The major pathways overrepresented across pseudotime included angiogenesis, Wnt/β-catenin signaling, and cancer stemness (Fig. 3t). The key gene expression changes across pseudotime were also identified through Geneswitches analysis (Fig. 3v). The change in SNAI1 expression closely correlates with CSC markers (SIX2, MSX2, and GSC) and Wnt/β-catenin signaling markers (HDAC11, SKP2) (Fig. 3u and Figure S4j-n).

**Fig. 4:**
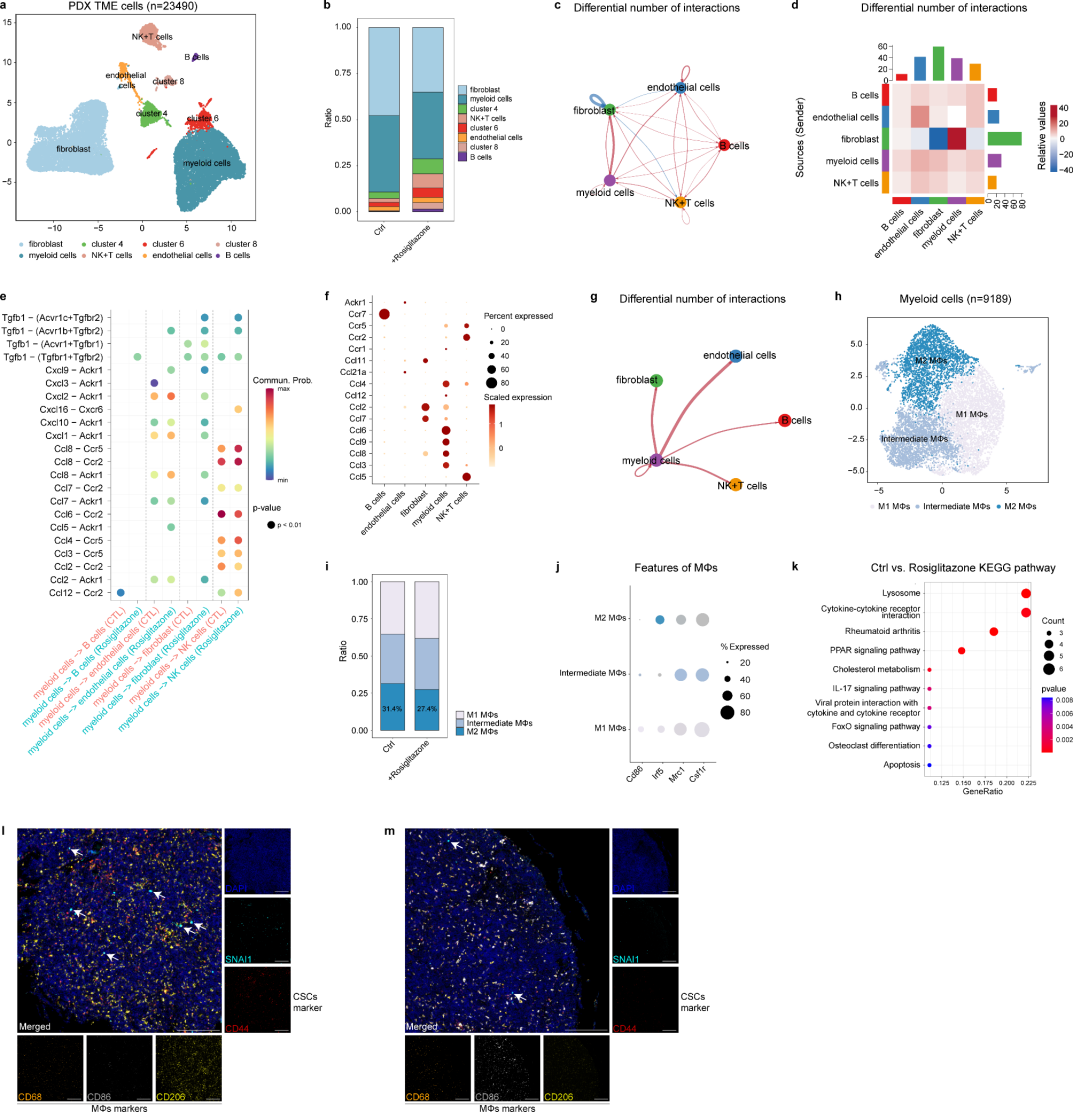
Evaluation of the impact of the application of the SNAI1 inhibitor on the tumor microenvironment through scRNA-seq. **a**. UMAP plot representing non-tumor mouse cells (dots) derived from PDX models of thymic squamous cell carcinoma, with color-coding based on global cell types. **b**. Relative cellular composition derived from the UMAP plot of non-tumor mouse cells treated with Rosiglitazone (SNAI1 inhibitor) or vehicle. **c**. Hierarchical plot from CellChat analysis showing the differential number of ligand-receptor interactions between murine microenvironment cells. **d**. Heatmap plot from CellChat analysis showing the differential number of ligand-receptor interactions between murine microenvironment cells. **e**. Dot plot showing significant chemokine-chemokine receptor pairs contributing to the signaling between myeloid cells and other cell types. **f**. Bubble plot showing significant chemokines contributing to signaling between different cell types. **g**. Hierarchical plot from CellChat analysis showing the differential number of ligand-receptor interactions between myeloid cells and other cell types. h-i. UMAP plot (**h**) and relative cellular composition (**i**) of mouse myeloid cells from PDX treated with Rosiglitazone or vehicle, color-coded by subtypes. **j**. Bubble plot of the mRNA expression levels of well-known markers for myeloid cells, including Cd86, Irf5, Mrc1, and Csf1r. **k**. KEGG pathway enrichment analysis of the DEGs in myeloid cells between the Rosiglitazone (SNAI1 inhibitor) and vehicle groups. **g**-**h**. mIHC images displaying the expression of SNAI1, CSCs marker (CD44), and myeloid cell markers (CD68, CD86, and CD206) in the TET tissue microarray, along with their merged image (Scale bars: 200 μm)

Similar to the study conducted by Lee et al. [25], we analyzed corresponding scRNA-seq data of the mouse TME cells to elucidate the effect of SNAI1 inhibitor on the TME. A total of 23,490 cells were identified from these two experimental groups (Fig. 4a, Fig. S5a–c). Unsupervised clustering unveiled the existence of murine TME components, comprising myeloid cells, fibroblasts, B cells, natural killer cells, and T cells (Fig. 4b). To better understand cell-cell interactions in the TME, we performed CellChat analysis. We first analyzed the differential number and interaction strength between different cell types (Fig. 4c-d, Fig. S5d–i). Since crosstalk between myeloid cells and other cell types was found to occur more frequently (Fig. 4g, Fig. S5k), we initially focused on the myeloid cells. The crosstalk between myeloid cells and other cell types primarily occurs through classic chemokines and chemokine receptors (Fig. 4e-f, Fig. S5j). We subsequently extracted all myeloid cells (*n* = 9189) and annotated the subgroups (Fig. 4h). Treatment with rosiglitazone led to a notable reduction in M2-like macrophages: with a 4% decrease in proportion, representing over 360 cells (Fig. 4i–k). Given that CSCs are reported to stimulate M1 to M2 macrophage polarization, the administration of rosiglitazone, by suppressing SNAI1 expression and inhibiting cancer stemness, could potentially reverse this polarization. To better analyze the changes in M2-like macrophages, we further performed single-sample gene set enrichment analysis (ssGSEA) based on the DEGs of M2-like macrophages between the two groups. An immune gene-related signature was subsequently constructed, and the scores generated in the rosiglitazone-treated group were significantly decreased (Fig. S5l).Moreover, we also examined the subgroup of fibroblasts. Significantly, rosiglitazone treatment augmented the population of cancer-associated fibroblasts, a subtype often correlated with increased tumor aggressiveness [26] (Fig. S5m,n). We also conducted an analysis of the DEGs between the treatment group and the control group. The KEGG analysis of the DEGs revealed enrichment in pathways, notably the PI3K/Akt signaling pathway, a classical pathway implicated in cancer mediation (Fig. S5o).

To corroborate the results uncovered by the scRNA-seq analysis, we conducted multiplex Immunohistochemistry (mIHC) using the tissue microarray. The intensity of CSCs marker (CD44^+^) positive cells was higher in the samples with a greater number of cells expressing SNAI1. Furthermore, the intensity of M1-like macrophage marker-positive (CD86^+^) cells was lower in the samples with a greater number of cells expressing SNAI1, while the intensity of M2-like macrophage marker-positive (CD206^+^) cells was higher in this group (Fig. 4l-m). These results demonstrate that SNAI1 has the potential to sustain cancer stemness and, consequently, promote the polarization of M1 to M2 macrophages.

### SNAI1 transcriptionally regulates the expression of PIK3R2 to promote tumor progression

**Fig. 5:**
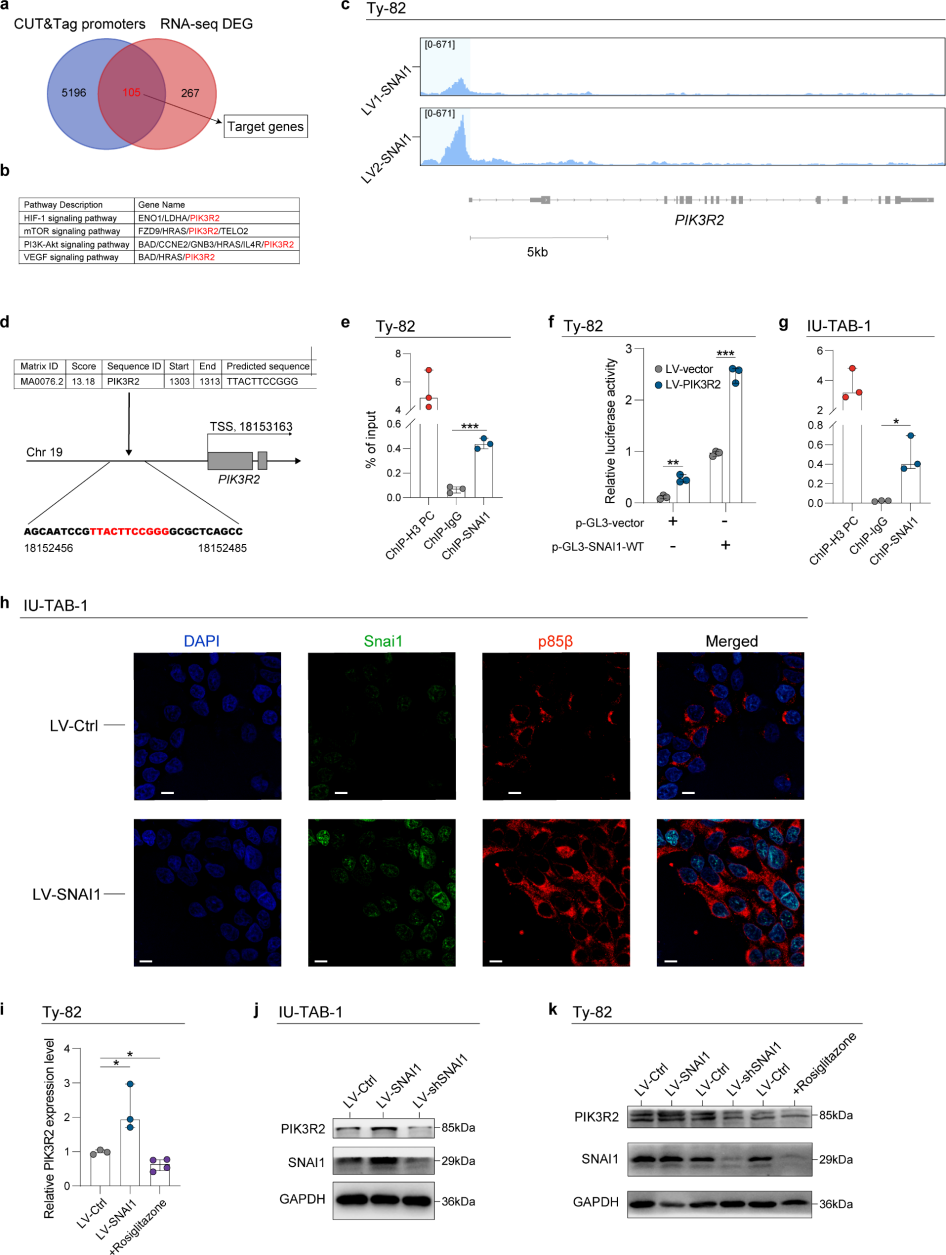
Selection of downstream targets of SNAI1. **a**. The Venn diagram illustrates the overlap between the annotated promoter regions of downstream genes identified through CUT&Tag sequencing and the DEGs identified through RNA-sequencing of the cell line. **b**. The outcomes of the KEGG pathway enrichment analysis for the overlapped genes and the key genes identified are presented in the results. **c**. The representative CUT&Tag peak tracks illustrate the binding peaks of SNAI1 to the upstream regions of PIK3R2 gene in Ty82 cells overexpressing SNAI1. Data from two replications are shown in the results. **d**. Highly probable SNAI1 binding sites within the PIK3R2 promoter region were identified using the JASPAR database. The illustration of the binding site is provided. **e**. The results of ChIP-qPCR depict the binding site on the PIK3R2 promoter with either the SNAI1 antibody, positive control (H3) or IgG in Ty82 cells (three independent samples). **f**. Bar plot showing the results of the luciferase reporter assay in two sets of Ty82 cells. **g**. The results of CUT&RUN-qPCR show the binding site on the PIK3R2 promoter with either the SNAI1 antibody, positive control (H3), or IgG in IU-TAB-1 cells (three independent samples). **h**. Representative images from the immunofluorescence colocalization assay performed on IU-TAB-1 cells overexpressing SNAI1 and control cells (Scale bars: 10 μm). **i**. qRT-PCR analysis indicates the relative PIK3R2 mRNA expression levels in Ty82 cells under various conditions, including those overexpressing SNAI1 (LV-SNAI1), treated with the SNAI1 inhibitor (Rosiglitazone), and the control group. **j**-**k**. Western blot analysis illustrating the protein expression levels of PIK3R2 and SNAI1 in TET cells transfected with different lentiviral vectors. * denotes *p* < 0.05, ** denotes *p* < 0.01, *** denotes *p* < 0.001

To comprehend the oncogenic mechanism in TETs, we conducted a further assessment of the target genes regulated by SNAI1. CUT&Tag sequencing was initially performed to assess the genomic interactions between SNAI1 and its downstream target genes. A total of 5,301 peaks were annotated as the promoter regions of the target genes. Furthermore, RNA sequencing was carried out to assess the DEGs between TET cells overexpressing SNAI1 and control cells. A total of 372 DEGs were identified (Fig. 5a). The overlapped 105 target genes of SNAI1 were subsequently analyzed through pathway enrichment analysis. The KEGG analysis unveiled several prominent oncogenic pathways, notably including the PI3K/Akt signaling pathways (Figure S6a). This is consistent with the previous results obtained through scRNA-seq analysis of the fibroblasts (Fig. S5o). Phosphoinositide-3-kinase, regulatory subunit 2 (PIK3R2) was identified as a hub gene within these oncogenic pathways (Fig. 5b). We first re-examined the results of the CUT&Tag sequencing and discovered that the distribution sites of SNAI1 were concentrated around the promoter region of PIK3R2 (Fig. 5c). To validate the sequencing results, we performed a promoter scanning in the JASPAR database and identified highly probable SNAI1 binding sites within the PIK3R2 promoter region (Predicted sequence: TTACTTCCGGG) (Fig. 5d). It overlaps with the binding sites identified through CUT&Tag. ChIP-qPCR analysis further validated the direct binding of SNAI1 to the promoter of PIK3R2 in Ty82 cells (Fig. 5e). A luciferase reporter assay was also conducted to validate the in vitro binding of SNAI1 to the PIK3R2 promoter region, using Ty82 cells (Fig. 5f). The CUT&RUN assay was also performed using IU-TAB-1 cell lines to demonstrate SNAI1 binding to the DNA of PIK3R2 (Fig. 5g). Additionally, the immunofluorescence colocalization assay revealed that Snai1 predominantly localizes in the nucleus, while p85β, encoded by PIK3R2, is mainly found in the cytoplasm in TET cell lines (Fig. 5h).

To investigate the clinical significance of PIK3R2 in TETs, we performed IHC staining on obtained TC tissues and adjacent normal thymic tissues. The staining score of PIK3R2 in TC tissues was confirmed to be higher than that in the corresponding normal counterparts (Figure S6b-d). We additionally confirmed that the overexpression of SNAI1 resulted in an elevation of the expression level of PIK3R2, whereas the knockdown of SNAI1 reversed this effect (Fig. 5i-k).

**Fig. 6:**
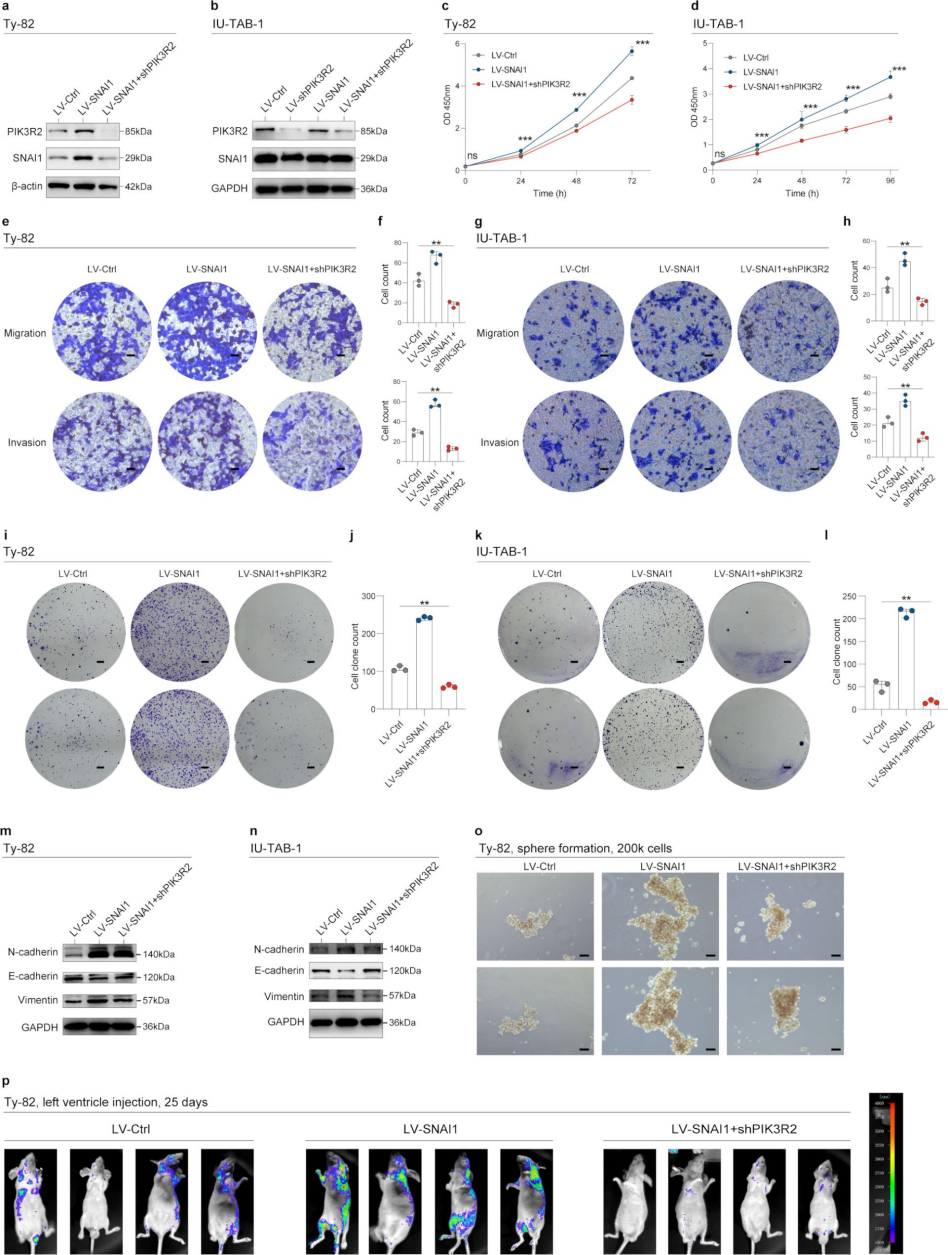
Validation of the oncogenic effects of PIK3R2 (also p85β). **a**-**b**. Western blot analysis illustrating the protein expression levels of PIK3R2 and SNAI1 in TET cells transfected with different lentiviral vectors. **c**-**d**. The effect of PIK3R2 knockdown on the proliferation of TET cells was evaluated using the CCK-8 assay, with absorbance at 450 nm measured at various time points. **e**-**h**. Transwell assays were conducted to assess the influence of PIK3R2 knockdown on the in vitro migration and invasion ability of TET cells, with representative micrographs presented (Scale bars: 200 μm). The quantification of cells attached to the lower surface of the chamber are also presented. **i**-**l**. The clone assay was utilized to assess the influence of PIK3R2 knockdown on the in vitro proliferation of TET cells. Representative micrographs are presented (Scale bars: 2 mm). The quantifications of cell clone spheres are also presented. **m**-**n**. Western blot analysis was conducted to evaluate the protein expression levels of EMT markers, including N-cadherin, E-cadherin, and Vimentin, in TET cells transfected with LV-Ctr, LV-SNAI1, or LV-SNAI1 + shPIK3R2. **o**. Representative images illustrate Ty82 cells transfected with LV-Ctrl, LV-SNAI1 or LV-SNAI1 + shPIK3R2 grown as spheres (Scale bars: 200 μm). **p**. Ty82 cells transfected with LV-Ctrl, LV-SNAI1 or LV-SNAI1 + shPIK3R2 were injected into the left ventricle of 5- to 6-week-old male wild-type BALB/c nude mice. Representative bioluminescent images illustrating TET cell metastases are presented. * denotes *p* < 0.05, ** denotes *p* < 0.01, *** denotes *p* < 0.001, and ns denotes not statistically significant

The knockdown of PIK3R2 also reversed the effects of SNAI1 overexpression in promoting the proliferation, migration, and invasion capabilities of TET cells (Fig. 6a-l). The expression levels of EMT markers decreased following the knockdown of PIK3R2 in TET cells (Fig. 6m-n). The sphere formation assay further confirmed that the knockdown of PIK3R2 attenuated tumor stemness (Fig. 6o). The metastatic abilities of TET cells were impaired following PIK3R2 knockdown (Fig. 6p). Taken together, these results suggest that SNAI1 functionally regulates the expression of PIK3R2, thereby promoting tumor invasiveness.

### PIK3R2 interacts with p-EphA2 and regulates the GSK3β/β-catenin pathway

**Fig. 7:**
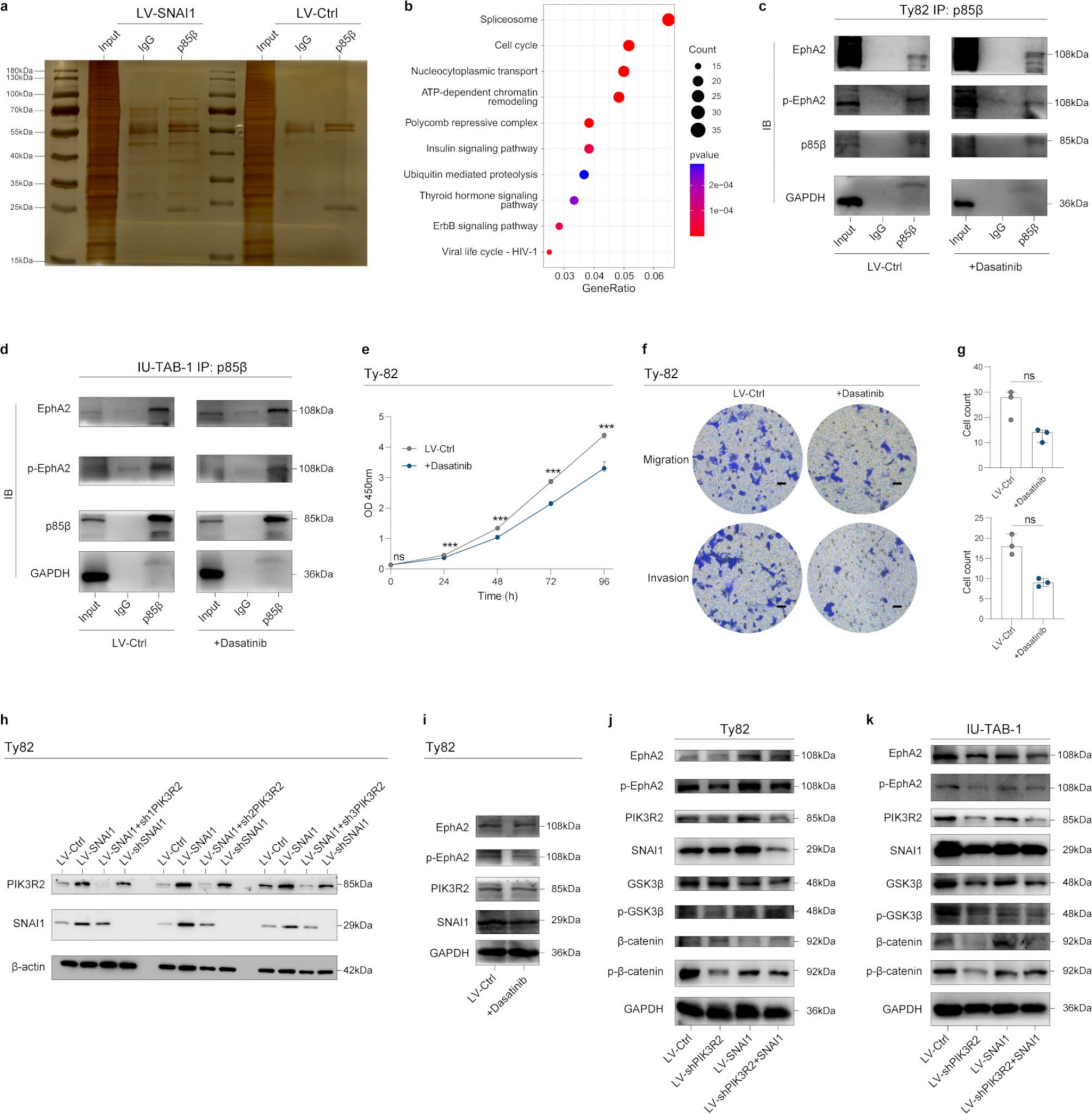
Exploration of the underlying mechanism was conducted through IP-MS, phosphoproteomics, and Co-IP. **a**. The results of silver staining demonstrate the proteins obtained through Co-IP using different antibodies, including p85β (encoded by PIK3R2) and IgG. **b**. KEGG pathway enrichment analysis was performed for the overlapped genes identified through mass spectrometry (MS) and phosphoproteomics. **c**-**d**. Results of co-immunoprecipitation (Co-IP) experiments showing the interaction between p-EphA2 and p85β in Ty82 cells (**c**) and IU-TAB-1 cells (**d**), both in the presence and absence of Dasatinib, an inhibitor targeting EphA2 phosphorylation at Ser897. **e**. The effect of administration of dasatinib on the proliferation of Ty82 cells was evaluated using the CCK-8 assay, with absorbance at 450 nm measured at various time points. **f**-**g**. Transwell assays were conducted to assess the influence of administration of dasatinib on the in vitro migration and invasion ability of Ty82 cells, with representative micrographs presented (Scale bars: 200 μm). The quantification of cells attached to the lower surface of the chamber are also presented. **h**. Western blot analysis was conducted to evaluate the protein expression levels of PIK3R2 and SNAI1 in Ty82 cells transfected with LV-SNAI1, LV-SNAI1 + shPIK3R2, or LV-shSNAI1. **i**. Western blot analysis was performed to assess the protein expression levels of EphA2, p-EphA2, PIK3R2, and SNAI1 in Ty82 cells treated with Dasatinib or vehicle. **j**-**k**. Western blot analysis was conducted to evaluate the protein expression levels of GSK3β/β-catenin signaling genes in TET cells transfected with LV-Ctrl, LV-shPIK3R2, LV-SNAI1, or LV-shPIK3R2 + SNAI1. *** denotes *p* < 0.001, and ns denotes not statistically significant

p85β, encoded by PIK3R2, serves as a crucial regulatory subunit of phosphatidylinositol 3-kinase (PI3K). Increased expression of p85β has been experimentally confirmed to induce phosphatidylinositol (3,4,5)-trisphosphate (PIP3) generation at the cell membrane, subsequently enhancing cell invasion [27]. To further explore the underlying mechanism of TET tumorigenicity, we performed immunoprecipitation-mass spectrometry (IP-MS) and phosphoproteomics in TET cells overexpressing SNAI1 (Fig. 7a,b). A total of 6,250 proteins and 2,979 phosphorylation sites were identified through the analysis, with the overlapping portion of these two groups consisting of 1,254 proteins. Amongst the identified proteins, our attention was drawn to the phosphorylated ephrin receptor A2 (EphA2) at Ser899. The EPH/ephrin (EPH/EFN) system exerts pivotal functions in cell proliferation, differentiation, and the activation of downstream tumorigenic signaling pathways [28]. The co-immunoprecipitation (co-IP) analysis validated the direct interaction between PIK3R2 and p-EphA2 in two TET cell lines (Fig. 7c-d). The administration of dasatinib, an inhibitor targeting EphA2 phosphorylation at Ser897, reduced the invasiveness of the TET cells (Fig. 7e-g). Interestingly the knockdown of PIK3R2 resulted in a decrease in the expression level of SNAI1 (Fig. 7h). However, with dasatinib administration, the protein expression levels of PIK3R2 and SNAI1 remained largely unchanged (Fig. 7i). Taken together, these results suggest the potential existence of a positive feedback loop involving SNAI1/PIK3R2 that contributes to the progression of TETs.

The downstream signaling pathway was further evaluated to ascertain the impact of the SNAI1/PIK3R2/p-EphA2 axis. As hypothesized, the downregulation of PIK3R2 resulted in a decrease in p-EphA2, phospho-GSK3β (Ser9), and phospho-β-catenin (Ser33/37/Thr41). Conversely, the overexpression of SNAI1 reversed these effects (Fig. 7j-k). These results suggest that the tumorigenic effects of TET cells may be modulated by the SNAI1/PIK3R2/p-EphA2 axis through the GSK3β/β-catenin signaling pathway.

## Discussion

**Fig. 8:**
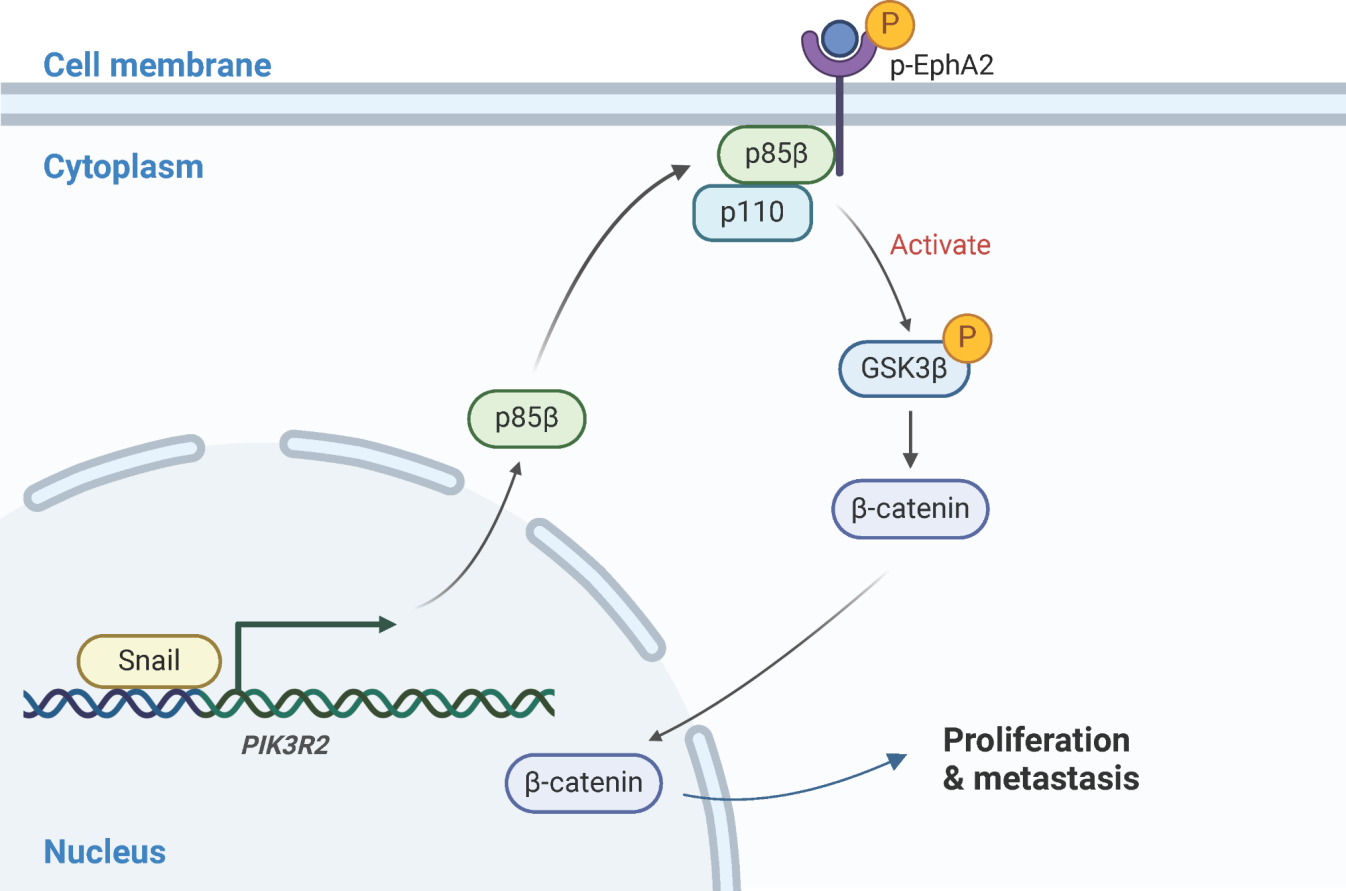
Schematic illustration of the SNAI1/PIK3R2/p-EphA2 axis in TETs. SNAI1 transcriptionally regulates the expression of PIK3R2, thereby promoting the synthesis of the p85β subunit. Subsequently, p85β is translocated to the cell membrane where it directly interacts with p-EphA2. Activation of the PI3K pathway and GSK3β/β-catenin signaling ensues, fostering tumorigenesis. The schematic graph was created using BioRender software (Ontario, Canada)

Thymic epithelial tumor (TET) is a heterogeneous and rare disease encompassing thymoma and thymic carcinoma (TC) [29, 30]. Approximately 10% of patients diagnosed with thymomas and 45% of patients with TCs do not survive beyond five years [31, 32]. Surgical resection, specifically total thymectomy, remains the cornerstone of treatment for TETs [2, 33, 34]. Despite the identification of molecular subtypes in the disease, current options for personalized medicine remain limited [35]. Recent clinical trials have highlighted the potential efficacy of two tyrosine kinase inhibitors, sunitinib and lenvatinib, in the treatment of chemotherapy-refractory TETs [7, 8]. However, the overall response rates remain unsatisfactory. Regarding the efficacy of immune checkpoint inhibitors (ICIs), both pembrolizumab and nivolumab have been employed in the non-first-line treatment of TETs [10, 11]. Only a small fraction of patients achieved a partial response, and the incidence of immune-related adverse events was relatively high. The potential for developing novel therapeutic strategies might lie in the exploration of the oncogenic mechanisms underlying the disease. To substantiate our hypothesis, we initially delved into the TCGA database to identify the central oncogene through bioinformatic analysis. Interestingly, SNAI1 emerged as one of the central transcriptional factors within the gene matrix. Subsequent experiments further corroborated these findings, affirming the role of SNAI1 in promoting epithelial-mesenchymal transition (EMT) and sustaining cancer stemness. The underlying mechanism was also investigated (Fig. 8). Our results provide a novel insight into a potential therapeutic strategy.

Although the histological subtype of TETs is commonly assumed to be associated with tumor invasiveness, it is noteworthy that all types of TETs possess the potential for disease progression and metastasis. Jain et al. uncovered that subtypes A and AB thymomas, traditionally regarded as benign diseases, may also exhibit malignant behavior, with 49% of patients experiencing recurrent/metastatic disease [36]. To gain a deeper understanding of this phenomenon, Radovich et al. endeavored to explore the molecular features of TETs [37]. They identified a substantial overexpression of a large microRNA cluster on chr19q13.42 in subtypes A and AB thymomas. The overexpression of this cluster was found to enhance the PI3K/AKT/mTOR pathway, thereby promoting tumorigenesis. This study affirms that molecular traits play a critical role in modulating tumor progression, complementing the influence of histological subtypes. Using multi-omics methods, Lee et al. subsequently developed a molecular classification system for TETs, identifying four distinct subgroups [4]. The molecular subgrouping was demonstrated to have a significant association with disease-free survival and the expression of PD-1. Building upon these preceding investigations, our objective was to identify the pivotal hub gene driving the disease and subsequently elucidate the downstream mechanism. Ultimately, we selected SNAI1, a central oncogenic transcription factor, as the focus of this study. We substantiated that the oncogenic effect is realized through the direct interaction between SNAI1 and PIK3R2. Overexpression of SNAI1 also heightened the GSK-3β/β-catenin signaling pathway, thereby facilitating tumorigenesis.

The phenomenon in which epithelial cells undergo a transformation into cells with a mesenchymal phenotype through specific procedures is referred to as epithelial-mesenchymal transition (EMT) [12]. This process is a common occurrence during embryonic development. EMT is also closely associated with increased tumor aggressiveness. Tumors undergoing EMT transformation often display a significantly worse long-term survival status [38]. Several highly conserved transcription factors play crucial regulatory roles in EMT. These transcription factors are selectively expressed in various types of tumor cells, thereby promoting the onset of EMT [13]. Snail1, a prominent member of the Snail superfamily, is a transcription factor that drives EMT. Elloul et al. demonstrated that Snail1 can suppress the expression of E-cadherin, contributing to poor clinical survival outcomes in metastatic ovarian and breast carcinoma [39]. Miyoshi et al. discovered a significant correlation between Snail1 and portal vein invasion and intrahepatic metastasis. Moreover, Snail1 was identified as an independent risk factor for early postoperative recurrence in hepatocellular carcinoma [40]. Studies investigating bladder carcinoma and gastric cancer have also confirmed that Snail1 induces EMT and exerts a cancer-promoting role [41, 42]. However, the investigation into the effects of Snail1 in TETs is still in its nascent stage. Wu et al. preliminarily validated a significant difference in Snail1 expression levels among different types of TETs [43]. The oncogenic role of Snail1 in TETs warrants further exploration. Huang et al. demonstrated that EphA2 promotes the EMT of gastric cancer cells through activation of Wnt/β-catenin signaling [44]. They also elucidated that the microRNA miR-302b regulates gastric cancer proliferation, invasion, and EMT by targeting EphA2 [45]. These results further highlight the critical role of EphA2 in promoting EMT. Masaoutis et al. elucidated that the expression of Eph type-A is associated with established prognostic parameters in TETs [46]. Overall, our study contributes to a better understanding of the tumorigenesis process of the disease.

We observed a particularly intriguing phenomenon during the experiments. It was validated that SNAI1 facilitates tumor progression and metastasis through the PIK3R2/p-EphA2 axis. The knockdown of PIK3R2 also resulted in the suppression of SNAI1’s protein level. Hence, we hypothesized the existence of a positive feedback loop within SNAI1 and PIK3R2. A feedback loop may exist between the functional proteins. Ji et al. illustrated a positive feedback loop between MYCN, a transcription factor, and ELAVL3, an RNA-binding protein [47]. These two genes thus contribute jointly to the progression of neuroendocrine prostate cancer. In contrast to the findings of this study, we identified PIK3R2, the regulatory subunit 2 of phosphatidylinositol 3-kinase, as the downstream target gene of SNAI1, a core transcription factor in TETs. Lin et al. found that Ephrin A4 (EFNA4), a ligand of the EPH family, directly interacts with EPHA2 and promotes its phosphorylation at Ser897, thereby enhancing cell proliferation and tumor metastasis in hepatocellular carcinoma [48]. They also discovered a PIK3R2/GSK3β/β-catenin positive feedback loop triggered by the abnormal expression of EFNA4. Although the oncogenic effects of the PI3K family are widely reported, the specific feedback effect on SNAI1 still requires elucidation.

Three notable limitations exist in this study. Firstly, it was demonstrated through Co-IP, MS, and phosphoproteomic assays that PIK3R2 directly interacts with p-EphA2. The phosphorylation site of EphA2 was identified as Ser899. Due to the unavailability of a commercially accessible antibody specific to phospho-EphA2 (Ser899) and the potential for bias in the mass spectrometry assay, we replaced it with an antibody designed for phospho-EphA2 (Ser897). Although the residue numbers for these two sites are closely situated, as indicated by PhosphoSitePlus v6.7.1.1 (Cell Signaling Technology), additional investigations are necessary to confirm these findings. Secondly, further exploration is needed to elucidate the underlying mechanism of the positive feedback loop involving SNAI1 and PIK3R2. Thirdly, as the tissue microarray included only two samples of type B3 thymoma and four samples of TC, the IHC assay based on this microarray may lead to potential misinterpretations. Future studies specifically comparing the invasiveness of type B3 thymoma and TC are still warranted.

## Conclusions

In summary, SNAI1 has been identified and validated as a prominent transcriptional factor in TETs. It can promote epithelial-mesenchymal transition and maintain the cancer stem cell-like properties of TET cells. It can also stimulate M1/M2 macrophage polarization to facilitate tumor stemness. The oncogenic effect of SNAI1 is mediated through the PIK3R2/p-EphA2 axis. These findings contribute to a deeper understanding of the tumorigenic mechanism underlying TETs and provide insights for the development of novel therapeutic strategies.

## Electronic supplementary material

Below is the link to the electronic supplementary material.

Supplementary Material 1

## Data Availability

The datasets used and/or analyzed during the current study are available from the corresponding author on reasonable request.

## Abbreviations

IQR: Interquartile range
TC: Thymic carcinoma
TET: Thymic epithelial tumor
TCGA: The Cancer Genome Atlas Program
WGCNA: Weighted gene co-expression network analysis
dissTOM: Dissimilarity Topological Overlap Matrix
ME: Module eigengene
DEG: Differentially expressed genes
LASSO: The Least Absolute Shrinkage and Selection Operator
EMT: Epithelial-mesenchymal transition
CSC: Cancer stem cell
scRNA-seq: Single-cell RNA sequencing
PDX: Patient-derived xenograft
ROS: Reactive oxygen species
TME: Tumor microenvironment
KEGG: Kyoto Encyclopedia of Genes and Genomes
CUT&Tag: Cleavage under targets and tagmentation
CUT&RUN: Cleavage under targets and release using nuclease
PIK3R2: Phosphoinositide-3-kinase, regulatory subunit 2
SNAI1: Snail family transcriptional repressor 1
EphA2: EPH receptor A2
ChIP: Chromatin immunoprecipitation
IHC: Immunohistochemistry
pan-CK: Pan-cytokeratin
mIHC: Multiplex immunohistochemistry
IP-MS: Immunoprecipitation-mass spectrometry
EphA2: Ephrin receptor A2
co-IP: Co-immunoprecipitation
qRT-PCR: Quantitative real-time polymerase chain reaction
SDS-PAGE: Sodium dodecyl-sulfate polyacrylamide gel electrophoresis
CCK-8: Cell counting kit-8
FC: Flow cytometry
RNA-seq: RNA sequencing
TPM: Transformed transcripts per kilobase million
GSK-3β: Glycogen synthase kinase-3 beta
PI3K: Phosphatidylinositol 3-kinase
