# Supplementary material for: SNAI1 promotes epithelial-mesenchymal transition and maintains cancer stem cell-like properties in thymic epithelial tumors through the PIK3R2/p-EphA2 Axis
Source: J Exp Clin Cancer Res. 2024 Dec 19;43:324. doi: 10.1186/s13046-024-03243-0 (PMC11657537; doi:10.1186/s13046-024-03243-0)

**Title: SNAI1 Promotes Epithelial-Mesenchymal Transition and Maintains Cancer Stem Cell-like Properties in Thymic Epithelial Tumors through the PIK3R2/p-EphA2 Axis**

Supplementary material

Supplementary Tables S1-2

Supplementary Figures S1-6

Table S1. Primer sequences employed for qRT-PCR in this study

| Gene | Forward primer sequence (5’ −> 3’) | Reverse primer sequence (5’ −> 3’) |
| --- | --- | --- |
| SNAI1 | TCGGAAGCCTAACTACAGCGA | AGATGAGCATTGGCAGCGAG |
| CD133 | GCCACCGCTCTAGATACTGC | TGTTGTGATGGGCTTGTCAT |
| CD44 | GAGCATCGGATTTGAGA | CATACTGGGAGGTGTTGG |
| PIK3R2 | AAAGGCGGGAACAATAAGCTG | CAACGGAGCAGAAGGTGAGTG |
| PIK3R2 (ChIP-qPCR) | AGCAATCCGTTACTTCCGGG | CAACGATCTCGACCCCTACG |
| GAPDH | GGAGCGAGATCCCTCCAAAAT | GGCTGTTGTCATACTTCTCATGG |

Table S2. Antibodies utilized in this study

| Antibodies | Source | Usage | Identifier |
| --- | --- | --- | --- |
| SNAI1 Polyclonal antibody | Proteintech | Immunoblot, IHC, mIHC | Cat. no., 13099-1-AP |
| Snail (C15D3) Rabbit mAb | Cell Signaling Technology | ChIP, Immunofluorescence, Immunoblot | Cat. no., 3879S |
| N-Cadherin (D4R1H) XP® Rabbit mAb | Cell Signaling Technology | Immunoblot | Cat. no., 13116S |
| Purified Mouse Anti-E-Cadherin | BD Biosciences | Immunoblot | Cat. no., 610181 |
| Vimentin (D21H3) XP® Rabbit mAb | Cell Signaling Technology | Immunoblot | Cat. no., 5741S |
| PI3 Kinase p85 Beta Monoclonal antibody | Proteintech | Immunoblot, IHC | Cat. no., 67644-1-Ig |
| Anti-PI 3 Kinase p85 beta antibody | Abcam | Co-IP | Cat. no., ab180967 |
| EPHA2 Monoclonal antibody | Proteintech | Immunoblot | Cat. no., 66736-1-Ig |
| Phospho-EPHA2 (Ser897) Antibody | Affinity Biosciences | Immunoblot | Cat. no., AF7279 |
| GSK3B Polyclonal antibody | Proteintech | Immunoblot | Cat. no., 22104-1-AP |
| Phospho-GSK3B (Ser9) Monoclonal antibody | Proteintech | Immunoblot | Cat. no., 67558-1-Ig |
| Beta Catenin Polyclonal antibody | Proteintech | Immunoblot | Cat. no., 51067-2-AP |
| Phospho-β-Catenin (Ser33/37/Thr41) Antibody | Cell Signaling Technology | Immunoblot | Cat. no., 9561S |
| Oval Cell Marker Antibody | Santa Cruz Biotechnology | Flow cytometry | Cat. no., sc-101863 |
| APC Mouse IgG2a, κ Isotype Control | Elabscience | Flow cytometry | Cat. no., E-AB-F09802E |
| PE Anti-Human/Mouse CD44 Antibody | Elabscience | Flow cytometry | Cat. no., E-AB-F1100D |
| Human/Rat OV-6 Antibody | R&D Systems | IHC | Cat. no., MAB2020 |
| Anti-pan Cytokeratin Mouse mAb | Servicebio | IHC | Cat. no., GB122053-100 |
| Anti-CD44 Rabbit pAb | Servicebio | mIHC | Cat. no., GB112054-100 |
| Anti-CD68 Rabbit pAb | Servicebio | mIHC | Cat. no., GB11067-100 |
| Anti-CD86 Rabbit pAb | Servicebio | mIHC | Cat. no., GB115630-100 |
| Anti-Mannose Receptor/CD206 Rabbit pAb | Servicebio | mIHC | Cat. no., GB115273-100 |
| Anti-beta Actin Mouse mAb | Engibody Biotechnology | Immunoblot | Cat. no., AT0001 |
| Anti-GAPDH Mouse mAb | Engibody Biotechnology | Immunoblot | Cat. no., AT0002 |

Figure S1. Supplementary results derived from the WGCNA and IHC outcomes of clinical specimens. a. Hierarchical clustering dendrogram of TCGA TET samples and the associated clinical trait heatmap. The color gradient in the clinical characteristics heatmap ranges from dark red to white, representing high to low levels of clinical characteristics, respectively. b. Hierarchical clustering dendrogram of major module eigengenes. The red line indicates a height cutoff of 0.3. c-e. Scatterplots depicting the gene significance for tumor differentiation (thymoma or TC, y-axis) versus module membership (x-axis) in three representative modules. f. Representative graphs illustrating IHC analysis of three clinical TET specimens diagnosed as thymic carcinoma, along with their matched adjacent normal tissues (Scale bars: 50 μm). SNAI1 polyclonal antibody (Proteintech, Cat. no. 13099-1-AP) was used in the IHC analysis. g. The final staining scores for these three specimens were evaluated by multiplying the staining intensity score by the positive cell ratio score. The scores were compared between tumor samples and the adjacent normal tissues. ns denotes not statistically significant.


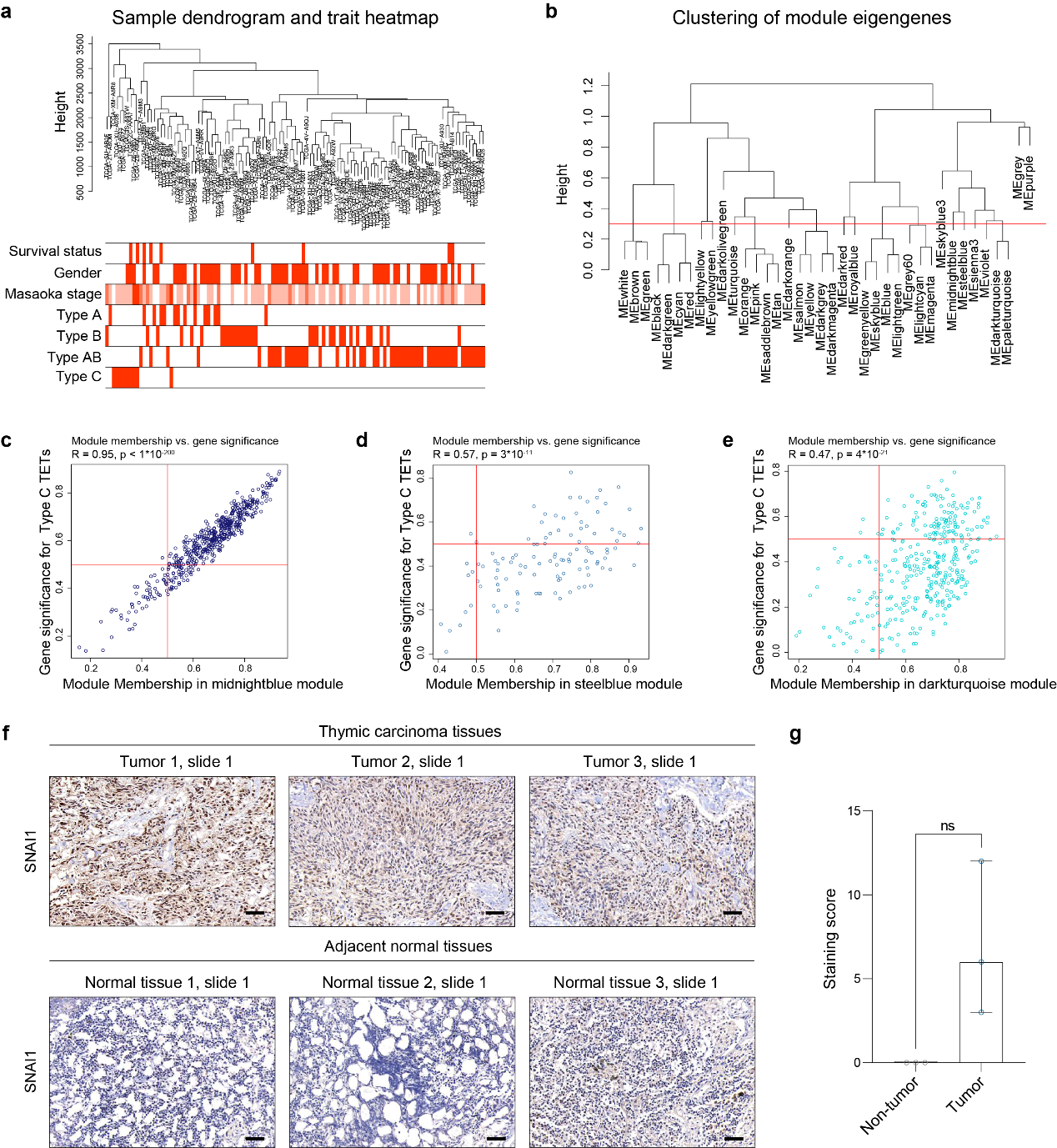


Figure S2. IHC results of SNAI1 and pan-CK using the TET tissue microarray. a. The tissue microarray included 54 samples, consisting of 46 TET samples, 2 thymic hyperplasia samples, and 6 normal thymic samples. The IHC results for SNAI1 are depicted in the graph. Four representative specimens’ staining results are magnified for detailed observation. b. The ultimate staining scores for SNAI1 were determined by multiplying the staining intensity score by the positive cell ratio score for all 54 specimens. The scores were compared among different subtypes and stages of the disease (TET only). c. The ultimate staining scores for pan-CK were determined. The scores were also compared. d. The IHC results for pan-CK are depicted in the graph. Four representative specimens’ staining results are magnified for detailed observation. * denotes *p* < 0.05, ** denotes *p* < 0.01, *** denotes *p* < 0.001, and ns denotes not statistically significant.


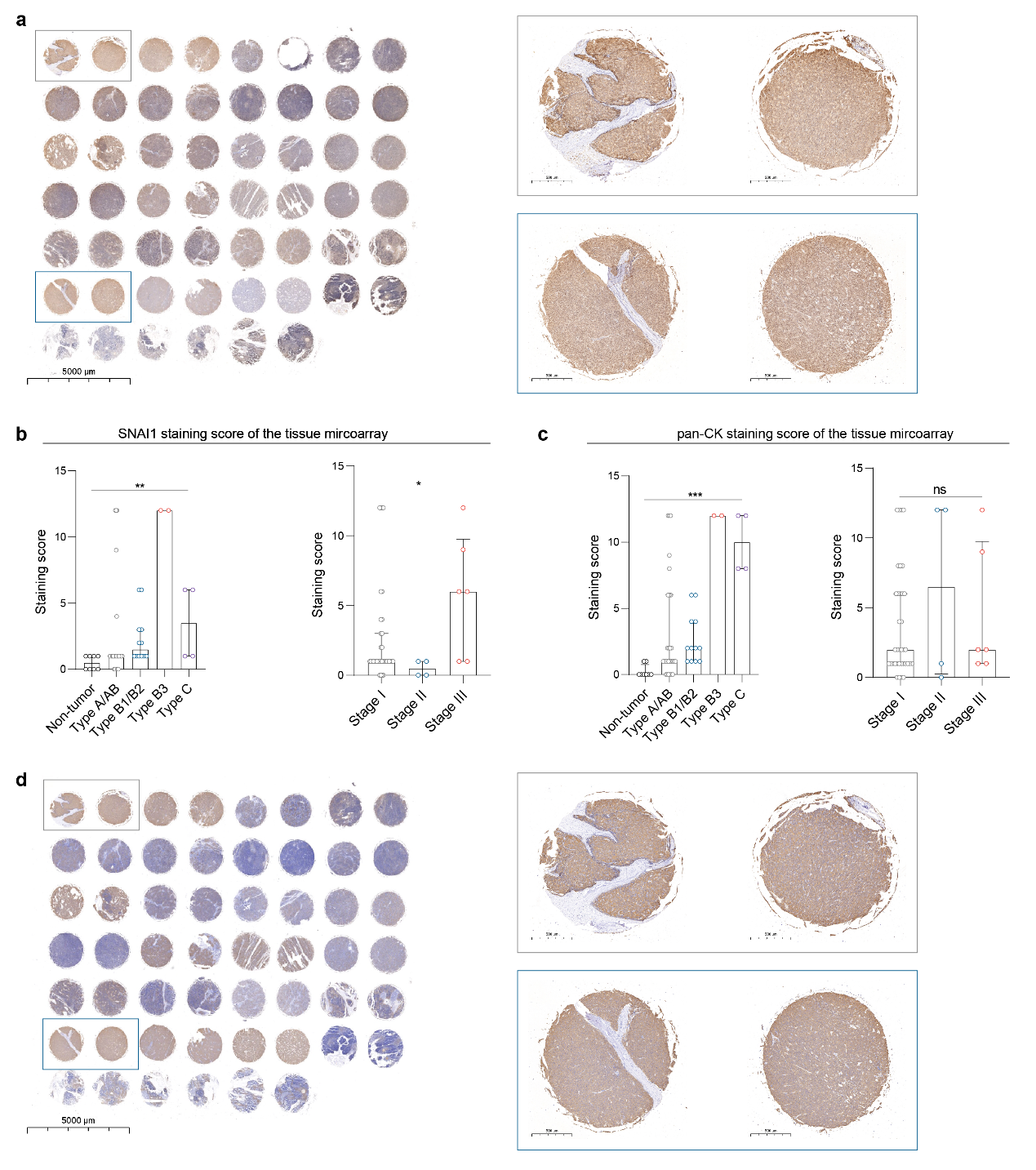


Figure S3. Supplementary results of findings from the in vitro validation of SNAI1’s oncogenic effects. a. qRT-PCR was employed to assess the relative expression levels of SNAI1 and CSC markers (CD133 and CD44) in Ty82 cells, comparing adherent cells to those in CSC spheres. b. qRT-PCR was employed to evaluate the relative expression levels of SNAI1 and CSC markers (CD133 and CD44) in CSC spheres, comparing cells transfected with LV-SNAI1 and LV-Ctrl. c. The IHC results for OV6, a CSC marker, are illustrated in the graph using the TET tissue microarray. Four representative specimens’ staining results are magnified for detailed observation. d. The ultimate staining scores for OV6 were determined by multiplying the staining intensity score by the positive cell ratio score for all 54 specimens. The scores were compared among different subtypes and stages of the disease (TET only). e. Flow cytometry analysis was conducted using CSC spheres cells transfected with LV-SNAI1 and LV-Ctrl. CSC spheres cells were subjected to incubation with antibodies in the following order: PE anti-human/mouse CD44, OV6 antibody, and APC mouse IgG2a for subsequent flow cytometry analysis. * denotes *p* < 0.05, ** denotes *p* < 0.01, *** denotes *p* < 0.001, and ns denotes not statistically significant.


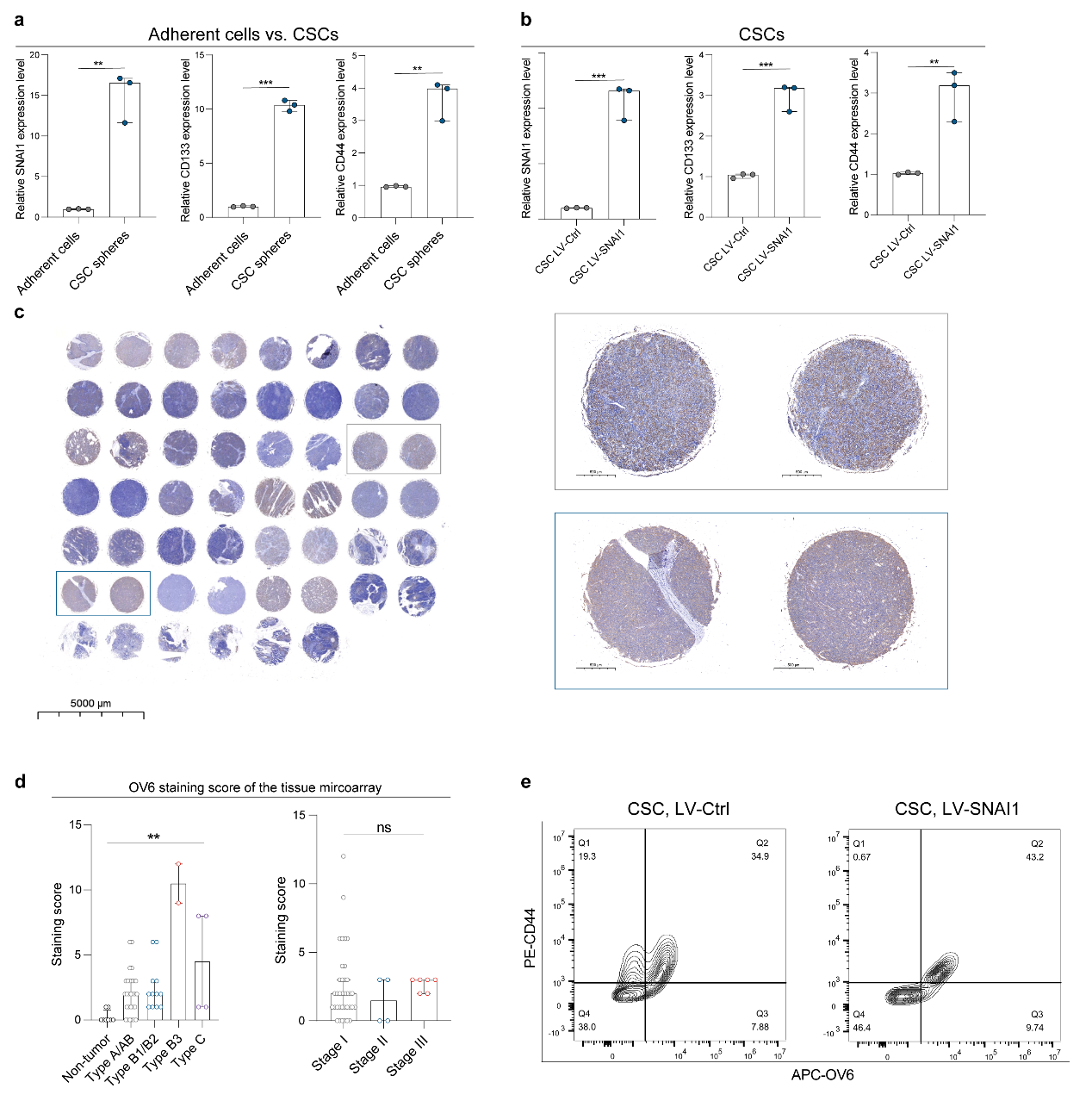


Figure S4. Supplementary results of scRNA-seq of TET cells. a. Boxplots demonstrating the normalized enrichment scores of cells in different states. b-d. Monocle plots demonstrating the differentiation trajectories based on monocle clusters, pseudotime, and gene expression status. e-g. t-SNE plot of tumor cells showing different CytoTRACE scores, subgroups, and different expression levels of SNAI1. h. Monocle plots demonstrating the differentiation trajectories based on CytoTRACE scores. i. Boxplot demonstrating CytoTRACE scores of different subgroups. j-n. UMAP projections of expression levels for CSC markers (SIX2, MSX2, and GSC) and Wnt/β-catenin signaling markers (HDAC11, SKP2). * denotes *p* < 0.05, ** denotes *p* < 0.01, and **** denotes *p* < 0.0001.


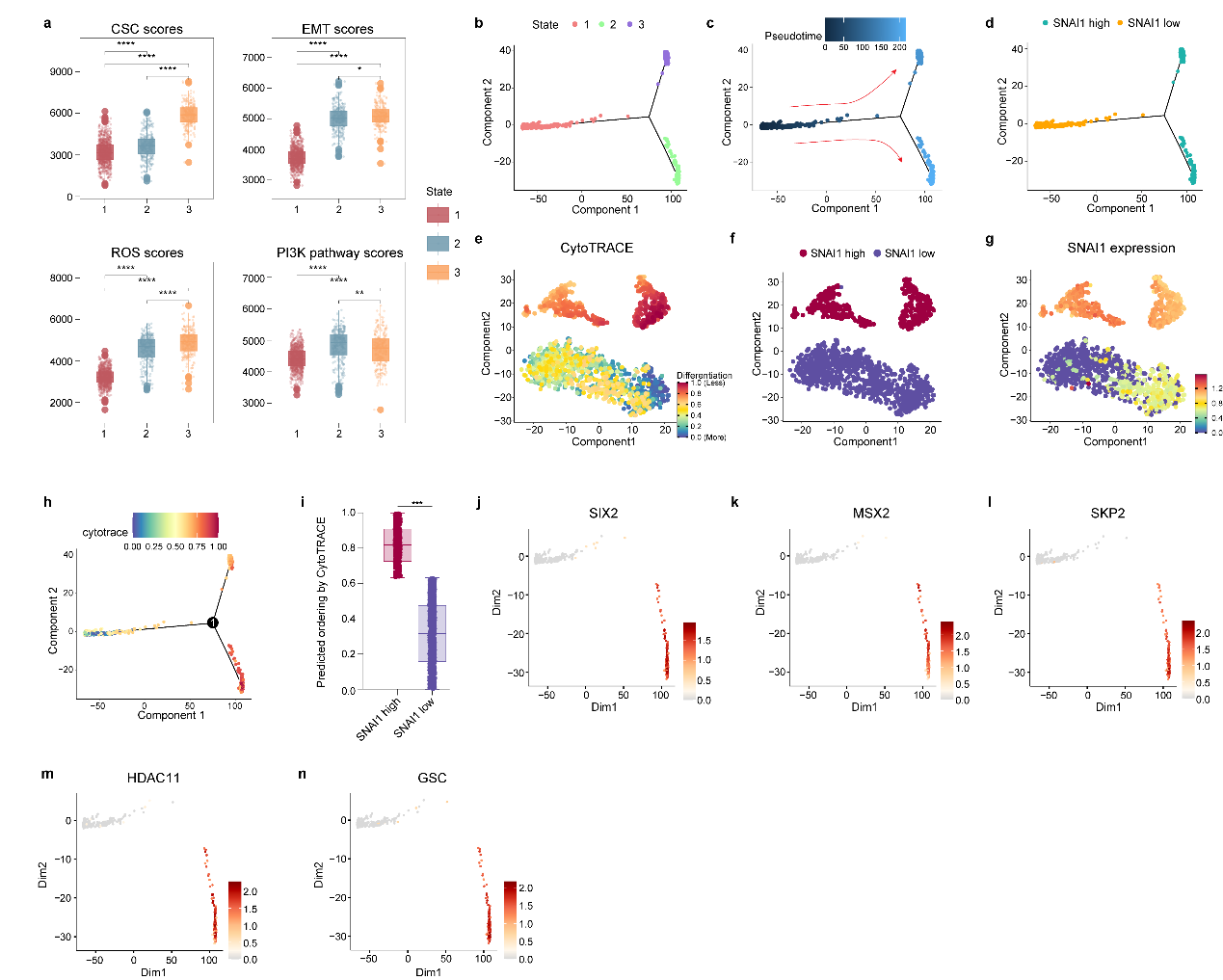


Figure S5. Supplementary results presenting the evaluation of the impact of the SNAI1 inhibitor on the tumor microenvironment through scRNA-seq. a. The “harmony” R package was employed for the integration of datasets originating from distinct treatment groups. b-c. Results of principal component analysis (PCA) and comparisons of embedding values between the Rosiglitazone (SNAI1 inhibitor) group and the control group after “harmony” integration. d. Hierarchical plot from CellChat analysis showing the differential strength of ligand-receptor interactions between murine microenvironment cells. e. Heatmap plot from CellChat analysis showing the differential strength of ligand-receptor interactions between murine microenvironment cells. f-g. Hierarchical plot from CellChat analysis showing the differential number (f) and strength (g) of ligand-receptor interactions between stromal and immune cells. h-i. Hierarchical plot from CellChat analysis showing the differential number of ligand-receptor interactions between stromal and immune cells in control (h) and Rosiglitazone (i) groups. j. Heatmap from CellChat analysis showing CCL signaling-specific interaction values between murine microenvironment cells. k. Hierarchical plot from CellChat analysis showing the differential strength of ligand-receptor interactions between myeloid cells and other cell types. l. Bar plot showing the immune gene-related signature scores between the rosiglitazone-treated and control groups. m-n. UMAP plot (m) and the relative cellular composition (n) of mouse fibroblast cells from PDX treated with Rosiglitazone or vehicle are presented, color-coded by subtypes. o. KEGG pathway enrichment analysis of the DEGs in fibroblast cells between the Rosiglitazone and vehicle groups.


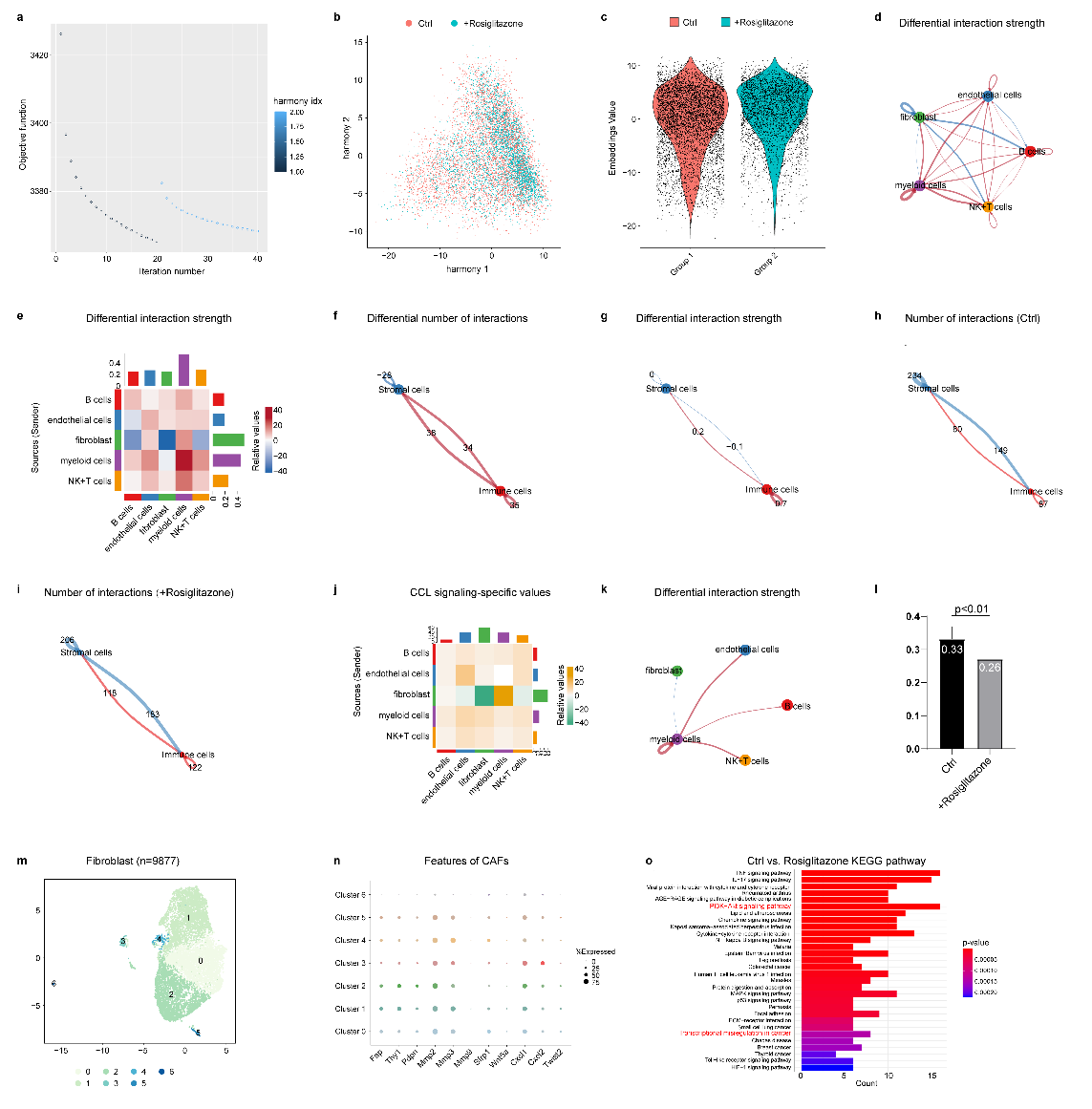


Figure S6. Supplementary results of additional findings regarding the confirmation of the oncogenic effects of PIK3R2. a. KEGG pathway enrichment analysis of the genes identified through CUT&Tag and the DEGs identified through RNA-sequencing. b. The final staining scores for these three specimens were evaluated by multiplying the staining intensity score by the positive cell ratio score. The scores were compared between tumor samples and the adjacent normal tissues. c-d. Representative graphs illustrating IHC analysis of PIK3R2 in three clinical TET specimens (each with two slides) diagnosed as thymic carcinoma, along with matched adjacent normal tissues. (Scale bars: 50 μm). ** denotes *p* < 0.01.


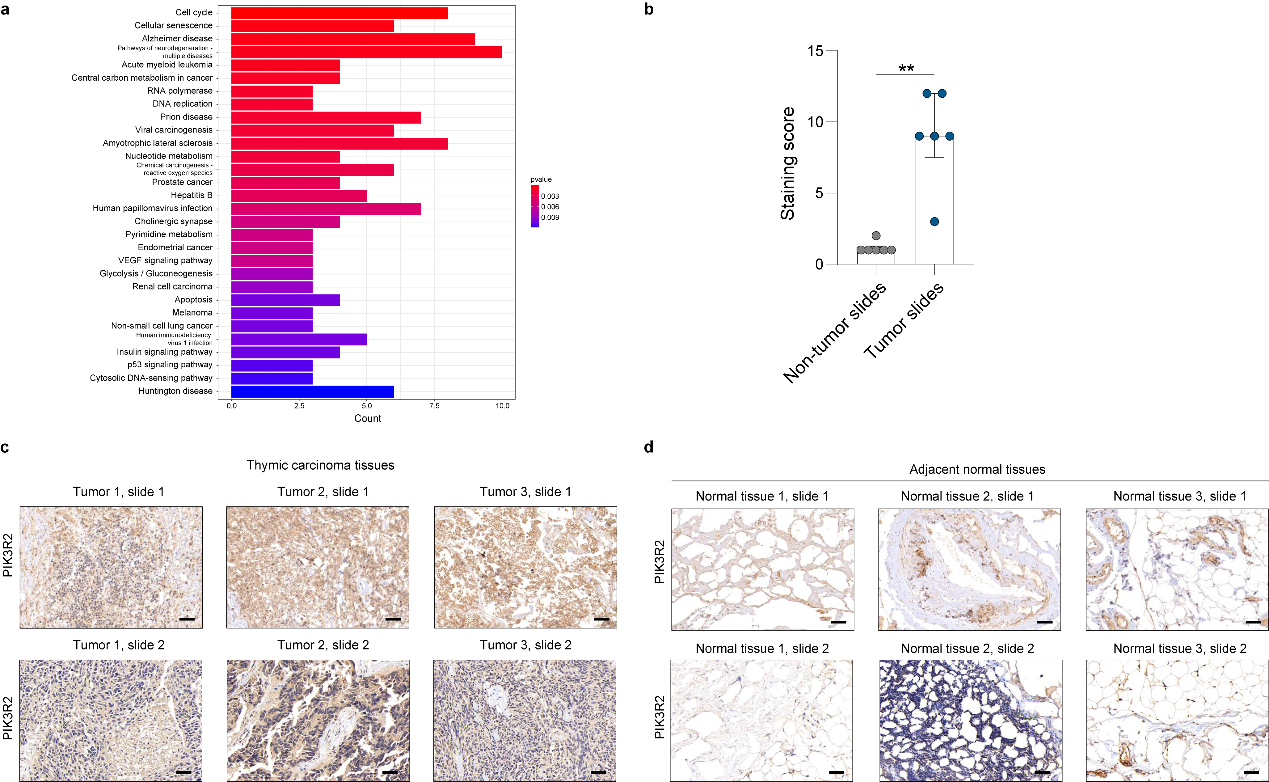

Supplement: Supplementary file 1 — Supplementary Material 1 [file 13046_2024_3243_MOESM1_ESM.docx]
